# Effect of plane of nutrition in early life on the transcriptome of visceral adipose tissue in Angus heifer calves

**DOI:** 10.1038/s41598-021-89252-x

**Published:** 2021-05-06

**Authors:** Kate Keogh, Alan K. Kelly, David A. Kenny

**Affiliations:** 1 Teagasc Animal and Bioscience Research Department, Teagasc Grange, Dunsany, Co Meath, Ireland; 2 School of Agriculture and Food Science, University College Dublin, Belfield, Dublin 4, Ireland

**Keywords:** Transcriptomics, Animal breeding

## Abstract

Adipose tissue represents not only an important energy storage tissue but also a major endocrine organ within the body, influencing many biochemical systems including metabolic status, immune function and energy homeostasis. The objective of this study was to evaluate the effect of an enhanced dietary intake during the early calfhood period on the transcriptome of visceral adipose tissue. Artificially reared Angus × Holstein–Friesian heifer calves were offered either a high (HI, n = 15) or moderate (MOD, n = 15) plane of nutrition from 3 to 21 weeks of life. At 21 weeks of age all calves were euthanized, visceral adipose harvested and samples subsequently subjected to mRNA sequencing. Plane of nutrition resulted in the differential expression of 1214 genes within visceral adipose tissue (adj. *p* < 0.05; fold change > 1.5). Differentially expressed genes were involved in processes related to metabolism and energy production. Biochemical pathways including Sirtuin signalling (adj. *p* < 0.0001) and the adipogenesis pathways (adj. *p* = 0.009) were also significantly enriched, indicating greater metabolic processing and adipogenesis in the calves on the high plane of nutrition. Results from this study identify novel genes regulating the molecular response of visceral adipose tissue to an improved plane of nutrition during early calfhood.

## Introduction

Adipose tissue depots including visceral, subcutaneous, intramuscular and intermuscular have evolved primarily as nutrient or energy storage tissues, providing a capacity to store energy in times of plenty and also providing fuel when food resources become insufficient^1,2^. However in addition to an important role in nutrient storage, adipose depots are also centrally involved in a number of additional physiological functions^3^. These are mainly derived by its key function as an endocrine organ, indeed adipose tissue is now recognised as the body’s largest endocrine organ, controlling many aspects of systemic physiology by secreting hormones, lipids, cytokines as well as other factors^4–6^. The peptide and non-peptide regulatory molecules secreted by adipose tissue elicit and control a wide variety of biological actions throughout the body including those associated with appetite, glycaemic regulation, insulin sensitivity, ageing, immune function, body temperature and reproductive function^1^. However, although there are several depots throughout the body, adipose deposition is not homogeneous^7^, with specific depots developing ahead of others during both fetal and post-natal developmental stages^7,8^. For example the formation of adipocytes commences during mid-gestation in beef cattle^9^, with adipocytes of the visceral fraction first detected followed by subcutaneous, intermuscular and then intramuscular deposition^2^. The same order of deposition is also apparent during post-natal development, with visceral deposition occurring during the early post-natal stage and the formation of subcutaneous adipocytes occurring slightly later, into the early weaning stage^2^. Moreover although each adipose fraction is essential to both energy storage and the organs endocrine function, varying depots may have specific discrete functions. For example, adipocytes within the visceral component are known to be particularly metabolically active^10^, which may be a direct consequence of these depots being located in close proximity to major metabolically active organs^11^.

An improved nutritional status for calves during early life has been shown to induce positive concurrent and latent effects for many economically important traits. For example, studies have shown improved lifetime growth, carcass composition and reproductive development as a consequence of enhanced metabolic status during early calf-hood^12,13^. This is particularly evident within the dairy industry whereby calves may be offered an elevated plane of nutrition during the first two to three months of life. Calves that undergo this type of dietary regimen typically display greater pre-weaning growth rates^14,15^, with heifers also having greater potential for subsequent milk production later in life^16,17^. Furthermore, in addition to the benefits for enhanced milk production, improved nutrition during this specific developmental window may also have subsequent beneficial effects on carcass composition^18^ and reproductive function^13^ of both male and female cattle. The effects of enhanced early life plane of nutrition may be most apparent within the adipose tissue depots as a means to develop energy stores early in the life-cycle. Indeed, the extent of adipose tissue development is dictated by the differentiation of stem cells into mature adipocytes^19^, and it has been postulated that the period in life for the differentiation of stem cells into adipocytes is particularly potent during early post-natal development^20,21^. Furthermore given the key endocrine role of adipose tissue, plane of nutrition may affect the ontogeny of adipogenesis, subsequently leading to the secretion of adipose derived regulatory molecules allowing for cross-talk between adipose tissues and other organs throughout the body, consequently influencing peripheral bodily functions^22^.

Thus the objective of this study was to evaluate the effect of an enhanced dietary intake during the early-life period on the transcriptional profile of visceral adipose tissue in heifer calves compared to contemporaries fed a typical moderate plane of nutrition. The visceral depot was targeted due to its known development during the early post-natal period^2^, as well as its apparent greater metabolic activity^10^. More specifically our study was focused on omental visceral adipose due to its reported greater metabolic activity compared to other depots^23–25^. Moreover, research into adipose tissue development in calves has shown that omental adipose is more responsive to variations in energy balance during the post-natal period^23^.

## Materials and methods

### Animal model

Tissue samples used in this study were derived from a larger study aimed at uncovering the impact of plane of nutrition during early calfhood on the physiological and molecular control of sexual development in the heifer calf^26^, the background experimental design is only briefly described here. Thirty Angus × Holstein–Friesian heifer calves with a mean (± SD) age and bodyweight of 19 (± 4) days and of 51.2 (± 7.8) kg, respectively, were blocked on age, bodyweight and sire and allocated within block to one of two dietary plane of nutrition groups: High (HI, n = 15) or Moderate (MOD, n = 15). Daily dietary allowances were formulated to support target average growth rates of > 1.2 kg/day and 0.50 kg/day for the HI and MOD nutritional treatments, respectively, until the calves reached 21 weeks of age. All calves were individually offered milk replacer and concentrate from the beginning of the trial up until weaning, using an electronic feeding system. Calves within the HI group were offered a milk feeding plan as follows: Stage I (days 0–30), 10 l of reconstituted milk replacer; Stage II (days 30–35), 10 l of reconstituted milk replacer gradually reduced to 6 l; Stage III (days 35–42), 6 l of reconstituted milk replacer and; Stage IV (days 42–56), 6 l of reconstituted milk replacer gradually reduced to 0 l. Moderately-fed calves (MOD) were offered a milk feeding plan as follows: Stage 1 (days 0–50) 4 l of reconstituted milk replacer; Stage II (days 50–56), 4 l of reconstituted milk replacer gradually reduced to 0 l. Milk replacer (20% fat and 26% protein) was reconstituted to 15.0% solids. Additionally, HI calves were offered concentrate ad libitum, whilst MOD calves received a stepped-up allowance, peaking at a maximum of 1 kg of concentrate per day during the week of weaning. Hay was also provided as a source of roughage (250 g/hd/day) and all calves also had ad libitum access to water. During the post-weaning phase of the trial, HI calves were offered concentrate ad libitum, whilst MOD calves were offered 1 kg of concentrate per day. Both treatment groups were offered hay to appetite during the post-weaning phase. Throughout the trial, all calves were weighed regularly on a weekly basis. At 21 weeks of age (145 ± 3 days), all calves were euthanized.

### Tissue isolation

Visceral adipose tissue was collected from the same site within the omental fraction from all heifers following euthanization. All instruments used for tissue collection were sterilised and treated with RNAzap prior to use. Tissue samples were washed in DPBS and subsequently snap frozen in liquid nitrogen. Tissue samples were subsequently stored at -80° C pending further processing.

### RNA isolation and RNA sequencing

Total RNA was isolated from all samples using the Qiagen RNeasy Plus Universal kit (Qiagen, UK) in accordance with the manufacturer’s instructions. Following isolation, RNA samples were quantified on the Nanodrop spectrometer, and RNA quality was assessed on the Agilent Bioanalyzer using the RNA 6000 bioanalyzer kit. Only samples with RNA integrity number (RIN) values greater than 8 were used for subsequent RNA-sequencing. cDNA libraries were prepared from 1 µg of total RNA for each sample using the Illumina Truseq stranded mRNA kit (Illumina, San Diego, CA, USA). Briefly, using 1 µg of total RNA as starting material for each sample, mRNA was purified and subsequently fragmented. SuperScript II Reverse Transcriptase (Applied Biosystems Ltd.) was used for first strand cDNA synthesis from the purified mRNA, with the second strand of cDNA synthesized using components of the Illumina Truseq kit. Following ligation of sequencing adapters, cDNA libraries were then enriched through PCR. Final cDNA libraries were then validated using the DNA 1000 Nano Lab Chip kit on the Agilent Bioanalyzer 2100, ensuring that library fragment size was ~ 260 bp and library concentration was > 30 ng/µl. Sequencing of cDNA libraries was then undertaken on an Illumina Novaseq platform employing 150 bp paired-end sequencing.

### Bioinformatic analysis

Resultant reads following RNA-sequencing were first checked for quality using Fastqc (version 0.11.7). Cutadapt software (version 1.18.8) was then used to remove sequencing indexing adapters as well as any low quality reads. Trimmed sequencing reads were then aligned to the bovine reference genome (UMD3.1) using the Spliced Transcripts Alignment to a Reference (STAR) aligner (version 2.5.2.b). Within STAR the quantmode function was utilized to quantify the number of sequencing reads aligned to each gene. The Bioconductor software, EdgeR (version 3.20.9), was subsequently used to determine genes differentially expressed between heifers fed the HI compared with the moderate plane of nutrition (MOD). Any gene with less than one count per million in at least half the number of samples (n = 15) was removed from the analysis. Data were normalised across libraries using the trimmed mean of M-values normalisation method. The quantile-adjusted conditional maximum likelihood common and tagwise dispersions were used to estimate gene expression dispersion. Exact tests were used for the detection of differentially expressed genes between calves on the HI and MOD diets. Genes with a Benjamini–Hochberg false discovery rate of 5% and a fold-change greater than 1.5 were considered differentially expressed. The resultant list of differentially expressed genes was then submitted to Ingenuity pathway analysis (IPA, Qiagen)^27^ in order to assign biological annotation and undertake biological pathway analysis. In addition to analysing all differentially expressed genes together through IPA, individual analyses for both up- and down-regulated genes were also undertaken in order to determine biological pathways both activated and de-regulated as a consequence of enhanced early life nutrition.

### Ethics declaration

All procedures involving animals were approved by the Teagasc Animal Ethics Committee, and licensed by the Health Products Regulatory Authority (Licence Number AE19132/P061) in accordance with the European Union Directive 2010/36/EU. All procedures were in compliance with the ARRIVE guidelines.

## Results

### Animal performance

The effect of plane of nutrition on feed intake and growth related traits as well as on a comprehensive blood analyte characterisation is outlined in detail by Kelly et al.^26^ and is only briefly described here. When euthanasia was performed at 21 weeks of age, calves in the HI group were on average 76.6 kg heavier than their MOD contemporaries (*p* < 0.001) consistent with the planned design of the study. In total throughout the entire trial, HI calves consumed 56.6 kg/DM of milk replacer and 27.7 kg of concentrate, whilst MOD calves consumed 26.9 kg/DM of milk replacer and 34.3 kg/DM of concentrate. Cumulatively daily energy intake was 2.5 times higher for HI calves compared to MOD calves (*p* < 0.001). Mean growth rates over the entire experimental period (pre- and post-weaning) were 1.18 kg/d and 0.50 kg/d for HI and MOD calves, respectively (*p* < 0.001).

### RNAseq and bioinformatic analyses

In total 153 million reads were generated from sequencing 30 (15 HI and 15 MOD) cDNA libraries, resulting in an average read number of 50.9 million per sample. Following alignment of trimmed sequencing reads to the bovine genome, more than 89% of reads were aligned to the protein coding regions of the genome (percentage of reads mapped: 89.04–91.34%). Following removal of lowly expressed genes within EdgeR analysis, 13,047 genes were retained for differential expression analysis, which resulted in 1214 genes being identified as differentially expressed (adj. *p* < 0.05; fold change > 1.5) between heifer calves fed the high and moderate energy diets. More specifically, of these genes, 720 were up-regulated and 494 genes down-regulated in the calves on the high diet compared to the moderate energy diet. RNAseq data derived from the current study have been deposited within NCBI’s Gene Expression Omnibus and are available through accession ID GSE158128.

### Biological pathway analysis

**Figure 1:**
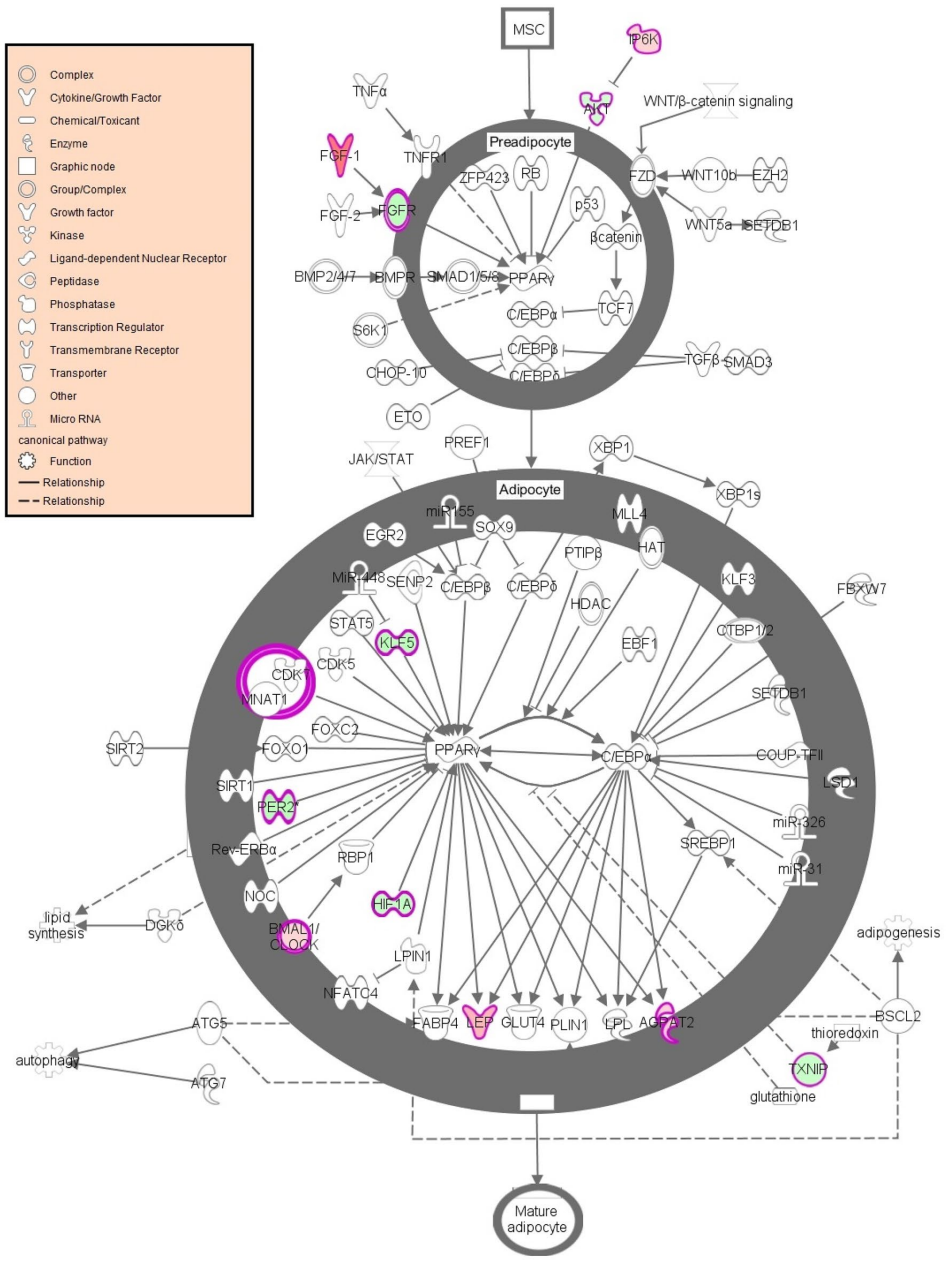
Aidpogenesis signalling pathway significantly enriched following altered plane of nutrition for the first 21 weeks of life in visceral adipose of heifer calves. Genes highlighted in red indicate genes that displayed greater expression in calves on the High (HI) plane of nutrition compared to calves fed a Moderate dietary intake (MOD). Genes highlighted in green indicate genes that displayed lower expression in HI calves compared to MOD calves. The Adipogenesis signalling pathway image was generated through the use of IPA (QIAGEN Inc., https://www.qiagenbio-informatics.com/products/ingenuity-pathway-analysis)24.

**Figure 2:**
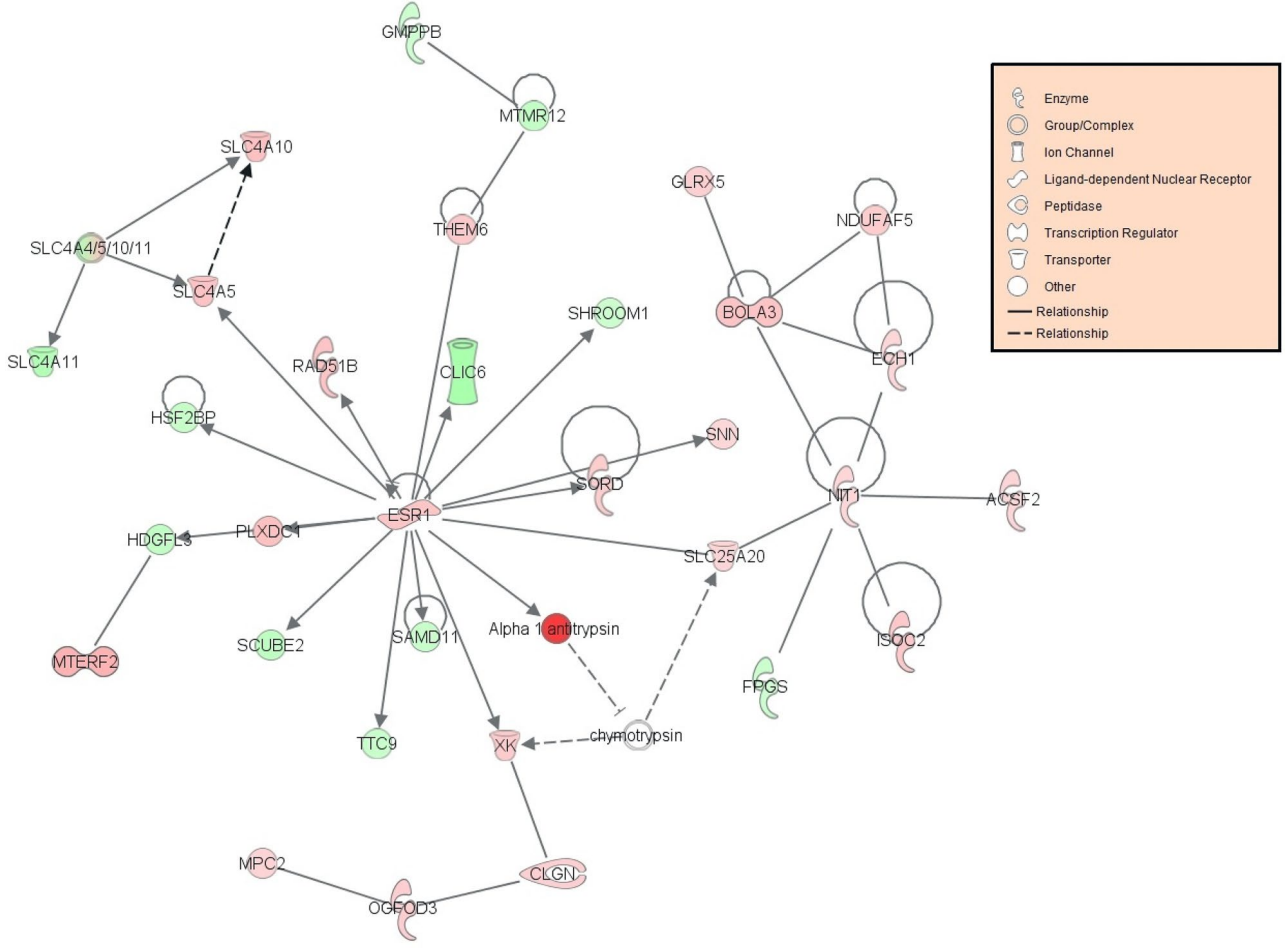
Molecular transport and the endocrine system network following altered plane of nutrition for the first 21 weeks of life in visceral adipose of heifer calves. The network (network 2) is displayed graphically as nodes (genes). The node colour intensity indicates the expression of genes; with red representing up-regulation and green, down-regulation in calves fed the High plane of nutrition compared to those fed a moderate plane of nutrition up to 21 weeks of age. The network image was generated through the use of IPA (QIAGEN Inc., https://www.qiagenbio-informatics.com/products/ingenuity-pathway-analysis)24.

**Figure 3:**
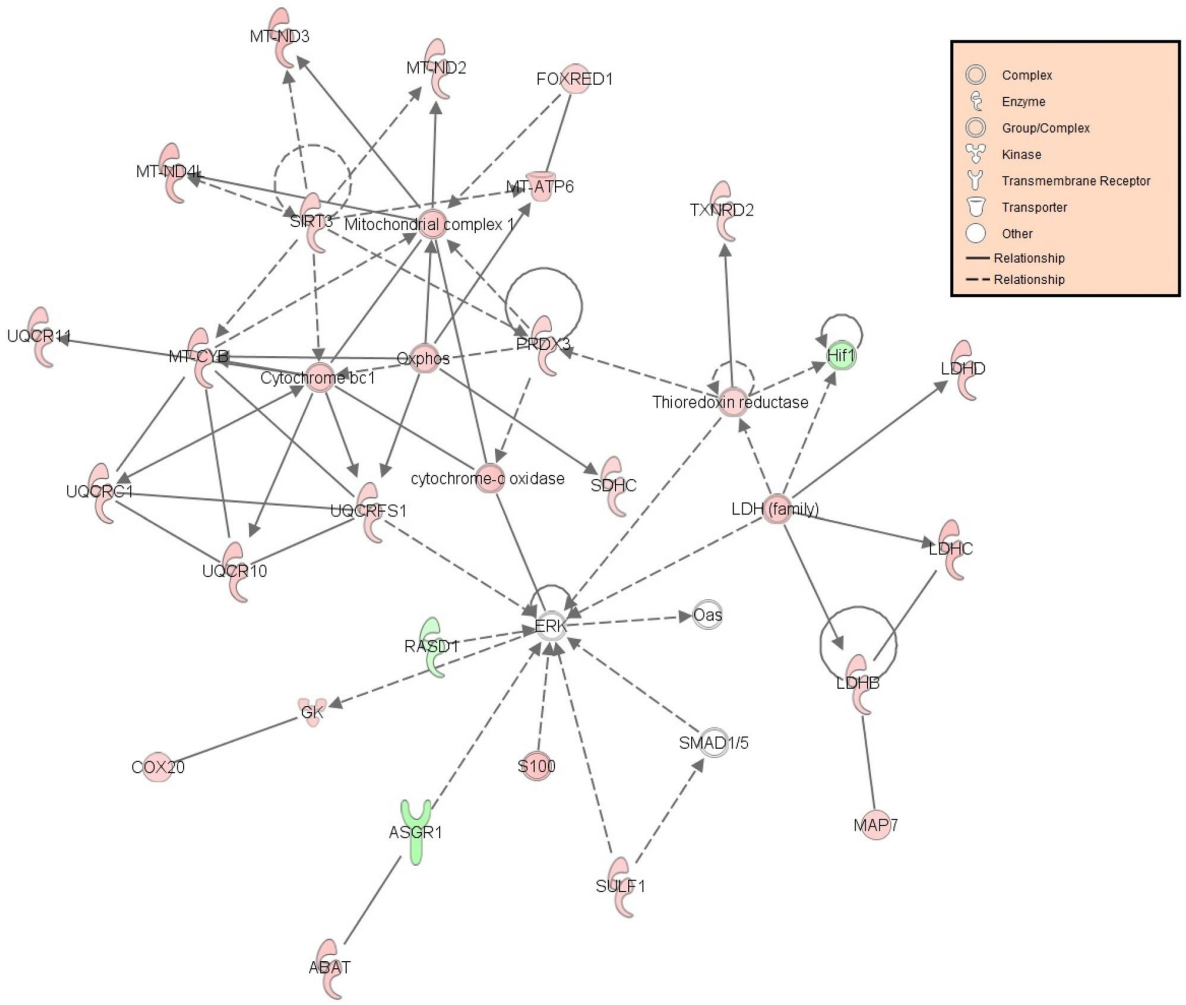
Small molecule biochemistry and metabolic processes network following altered plane of nutrition for the first 21 weeks of life in visceral adipose of heifer calves. The network (network 12) is displayed graphically as nodes (genes). The node colour intensity indicates the expression of genes; with red representing up-regulation and green, down-regulation in calves fed the High plane of nutrition compared to those fed a moderate plane of nutrition up to 21 weeks of age. The network image was generated through the use of IPA (QIAGEN Inc., https://www.qiagenbio-informatics.com/products/ingenuity-pathway-analysis)24.

Out of the 1214 genes identified as differentially expressed from EdgeR analysis, 928 genes were successfully mapped to a biological function or pathway within the Ingenuity Pathway knowledgebase. Mapped genes are listed in Supplementary Table S1. Biological functions affected by early life plane of nutrition, included those related to metabolism: lipid, carbohydrate and amino acid, as well as cellular signalling. A total of 96 biochemical pathways were found to be enriched (*p* < 0.05) in IPA based on the input list of differentially expressed genes. Enriched biological pathways affected by early life plane of nutrition are outlined in full in Supplementary Table S2 and include enriched pathways of interest such as Sirtuin signalling (adj. *p* < 0.0001) and adipogenesis (adj. *p* = 0.009). Fold change values of genes pertaining to these pathways of interest are presented in Tables 1 and 2, respectively. Additionally genes differentially expressed within the adipogenesis pathway are presented graphically in Fig. 1. Pathway analysis in IPA generated a number of biologically relevant networks including Network 2, related to molecular transport and the endocrine system and Network 12, which involved genes associated with small molecule biochemistry and metabolic processes. The full list of networks generated within IPA are outlined in Supplementary Table S3, with images for networks of interest including Network 2 and Network 12, presented in Figs. 2 and 3, respectively. We were particularly interested in the potential role of molecular processes within the adipose tissue in regulating reproductive function, thus genes identified as being affected by early-life plane of nutrition in adipose tissue and having an associated reproductive function are presented in Table 3. Finally enrichment of biological pathways based on up- and down-regulated genes are presented in Supplementary Tables S4 and S5, respectively. De-regulated pathways (down-regulated in HI calves) included IGF-1 (adj. *p* = 0.0089) and mTOR (adj. *p* = 0.0102) signalling. Activated pathways based on genes up-regulated in adipose tissue of HI calves included oxidative phosphorylation (adj. *p* < 0.0001), Sirtuin signalling (adj. *p* < 0.0001), and estrogen receptor signalling (adj. *p* = 0.0436).

**Table 1:** Genes mapped to the Sirtuin signaling pathway identified as differentially expressed in visceral adipose tissue between calves fed high or moderate energy diets during the early life period up to 21 weeks of age.

Gene ID	Gene Name	Fold Change^a^	FDR^b^
*ACLY*	ATP citrate lyase	4.067	5.36E−07
*ACSS2*	Acyl-CoA synthetase short chain family member 2	3.041	5.98E−06
*AKT1*	AKT serinethreonine kinase 1	− 1.515	0.000398
*ARNTL*	Aryl hydrocarbon receptor nuclear translocator like	1.586	0.0172
*G6PD*	Glucose-6-phosphate dehydrogenase	2.684	1.19E−07
*GOT2*	Glutamic-oxaloacetic transaminase 2	1.634	0.0182
*HIF1A*	Hypoxia inducible factor 1 subunit alpha	− 1.954	0.00512
*HIST1H4J*	Histone cluster 1 H4 family member j	− 1.762	0.0397
*LDHB*	Lactate dehydrogenase B	1.767	0.00103
*LDHC*	Lactate dehydrogenase C	2.285	0.00283
*LDHD*	Lactate dehydrogenase D	1.965	0.000112
*MAP1LC3A*	Microtubule associated protein 1 light chain 3 alpha	1.622	0.0313
*MT-ATP6*	ATP synthase F0 subunit 6	2.002	0.000422
*MT-CYB*	Cytochrome b	2.14	0.00139
*MT-ND1*	NADH dehydrogenase, subunit 1 (complex I)	1.984	0.000177
*MT-ND2*	MTND2	1.727	0.00409
*MT-ND3*	NADH dehydrogenase, subunit 3 (complex I)	2.518	2.24E−05
*MT-ND4*	NADH dehydrogenase, subunit 4 (complex I)	2.189	0.000069
*MT-ND5*	NADH dehydrogenase, subunit 5 (complex I)	1.972	0.00236
*MT-ND4L*	NADH dehydrogenase, subunit 4L (complex I)	2.364	0.00496
*MYC*	MYC proto-oncogene, bHLH transcription factor	− 2.287	8.77E−05
*NDUFA1*	NADH:ubiquinone oxidoreductase subunit A1	1.916	2.31E−05
*NDUFA2*	NADH:ubiquinone oxidoreductase subunit A2	1.687	0.00867
*NDUFA4*	NDUFA4, mitochondrial complex associated	1.666	0.0227
*NDUFA10*	NADH:ubiquinone oxidoreductase subunit A10	2.002	0.0018
*NDUFA13*	NADH:ubiquinone oxidoreductase subunit A13	1.783	0.0121
*NDUFAB1*	NADH:ubiquinone oxidoreductase subunit AB1	1.692	0.0399
*NDUFB3*	NADH:ubiquinone oxidoreductase subunit B3	1.521	0.0374
*NDUFS2*	NADH:ubiquinone oxidoreductase core subunit S2	1.719	0.00614
*NDUFS3*	NADH:ubiquinone oxidoreductase core subunit S3	1.659	0.0354
*NDUFS6*	NADH:ubiquinone oxidoreductase subunit S6	1.737	0.0249
*NDUFV1*	NADH:ubiquinone oxidoreductase core subunit V1	2.138	0.00159
*NQO1*	NAD(P)H quinone dehydrogenase 1	2.409	7.66E−08
*PCK1*	Phosphoenolpyruvate carboxykinase 1	2.351	0.00332
*PDHA1*	Pyruvate dehydrogenase E1 alpha 1 subunit	1.63	0.0214
*PGAM1*	Phosphoglycerate mutase 1	1.523	0.0123
*SDHA*	Succinate dehydrogenase complex flavoprotein subunit A	2.162	0.000183
*SDHC*	Succinate dehydrogenase complex subunit C	1.507	0.0155
*SIRT3*	Sirtuin 3	1.793	0.000281
*SLC25A4*	Solute carrier family 25 member 4	2.19	0.0102
*SOD1*	Superoxide dismutase 1	1.585	0.00044
*SOD2*	Superoxide dismutase 2	1.853	0.000523
*TIMM23*	Translocase of inner mitochondrial membrane 23	1.72	0.00399
*Tomm5*	Translocase of outer mitochondrial membrane 5	2.002	0.00326
*TUBA1B*	Tubulin alpha 1b	− 1.664	0.0242
*UQCRFS1*	ubiquinol-cytochrome c reductase, Rieske iron-sulfur Polypeptide 1	1.659	0.022

^a^Fold change values are up- or down-regulated in high energy diet group compared to the moderate energy diet group.

^b^False Discovery Rate.

**Table 2:** Genes mapped to the adipogenesis pathway identified as differentially expressed in visceral adipose tissue between calves fed high or moderate energy diets during the early life period un to 21 weeks of age.

Gene ID	Gene Name	Fold Change^a^	FDR^b^
*AGPAT2*	1-acylglycerol-3-phosphate O-acyltransferase 2	1.652	0.0139
*AKT1*	AKT serinethreonine kinase 1	− 1.515	0.000398
*ARNTL*	Aryl hydrocarbon receptor nuclear translocator like	1.586	0.0172
*FGF1*	Fibroblast growth factor 1	5.182	4.83E−15
*FGFR3*	Fibroblast growth factor receptor 3	− 2.088	0.00267
*GTF2H5*	General transcription factor IIH subunit 5	1.522	0.0214
*HIF1A*	Hypoxia inducible factor 1 subunit alpha	− 1.954	0.00512
*KLF5*	Kruppel like factor 5	− 2.21	0.00167
*LEP*	Leptin	2.757	7.02E−05
*PER2*	Period circadian regulator 2	− 2.257	0.0028
*PPIP5K1*	Diphosphoinositol pentakisphosphate kinase 1	1.619	0.00871
*TXNIP*	Thioredoxin interacting protein	− 1.703	0.0175

^a^Fold change values are up- or down-regulated in high energy diet group compared to the moderate energy diet group.

^b^False Discovery Rate.

**Table 3:** Genes involved in reproductive development identified as differentially expressed in visceral adipose tissue between calves fed high or moderate energy diets during the early life period up to 21 weeks of age.

Gene ID	Gene name	Fold Change^a^	FDR^b^
*ESR1*	Estrogen receptor 1	2.199	0.0109
*GPER1*	G protein-coupled estrogen receptor 1	1.889	0.000357
*HSD17B12*	Hydroxysteroid 17-beta dehydrogenase 12	1.891	0.017
*GREB1*	Growth regulating estrogen receptor binding 1	3.379	0.000068
*SRD5A1*	Steroid 5 alpha-reductase 1	2.097	3.1E−07

^a^Fold change values are up- or down-regulated in high energy diet group compared to the moderate energy diet group.

^b^False Discovery Rate.

## Discussion

The final extent of adipose tissue development in the body is determined by differentiation of stem cells into mature adipocytes with the early post-natal period particularly important for this effect^19^. Enhanced plane of nutrition during the early-life period in calves has been shown to not only enhance overall growth rates but also contributes to the development of adipose depots^28^. Moreover, enhanced early life nutrition has been shown to be beneficial for subsequent lifelong growth and production potential of an animal^29^. Lifelong benefits of enhanced nutrition and associated adipogenesis during the early life period may also potentially include enhanced lactogenesis potential^16^, earlier reproductive development^13^ and improved carcass composition^30^. Thus, again the objective of this study was to evaluate the effect of enhanced dietary intake during the early-life period on the transcriptional profile of visceral adipose tissue in heifer calves compared to contemporaries fed a typical moderate plane of nutrition. Additionally, our aim was to understand the cross-talk between greater adipogenesis and the subsequent potential endocrine actions of visceral adipose tissue. The visceral adipose fraction was targeted as visceral adipose is more metabolically active, displays greater sensitivity towards lipolysis and is more insulin resistant when compared to subcutaneous adipose^10^. However, despite clear differences in deposition and function of varying adipose depots, results from the current study focused on omental visceral adipose show clear comparisons with the subcutaneous adipose data of English et al.^28^. The latter of which was concerned with investigating the effect of enhanced nutrition in bull calves during early life. Commonality between both tissue depots was established not only through the common differential expression of genes (180 genes commonly differentially expressed, 172 following the same direction of effect across studies (Supplemental Table S1)) but also the common enrichment of biological pathways and functions across studies. Equally though, differentially expressed genes unique to particular tissue depots were also established, which may represent differential functions of the varying depots, or alternatively may be due to employment of two genders, with heifers known to display earlier physiological maturity when compared to bulls of similar developmental stage. Although differentially expressed genes from this study were subjected to pathway analyses consisting of all differentially expressed genes as well as individual up- and down-regulated gene lists, the remainder of this discussion will focus on the complete list of both up-and down-regulated genes together, due to the importance of analysing both inhibitory and activating genes within various biochemical pathways in unison.

### Metabolism and Sirtuin signalling

Unsurprisingly, the contrasting dietary regimens employed between the two treatment groups led to divergence in the expression of genes involved in metabolic processes and nutrient transport. This was particularly evident through the enrichment of biological pathways and functions involved in carbohydrate metabolism as well as lipid and amino acid synthesis and degradation pathways (Supplementary Table S2). Similarly, as a consequence of their higher plane of nutrition, the adipose tissue of HI calves also displayed greater expression of genes involved in oxidative phosphorylation and mitochondrial energy production. Up-regulation of oxidative phosphorylation genes in the HI group implies an overall greater derivation of energy derived from the ingested dietary nutrients, which could then subsequently result in more energy being available for anabolic purposes within the adipose tissue and concurs with previous finding from our group examining enhanced early life nutrition on the transcriptome of subcutaneous adipose tissue in bull calves^28^. Whilst we have also reported altered expression of genes involved in metabolism and energy production processes in skeletal muscle and liver tissues of Holstein Friesian bulls fed a moderately restricted diet in order to induce compensatory growth in comparison to a non-diet restricted contemporary group^31,32^. However results from the current study indicate that the enhanced energy production of the HI group may be regulated by Sirtuins, a family of signalling proteins that are involved in metabolic regulation^33,34^, mediated through the Sirtuin signalling pathway. Results from the current study showed that *SIRT3*, which encodes a mitochondrial localised SIRT protein was up-regulated in the adipose tissue of HI compared to the MOD calves. Differential expression of *SIRT3* is particularly interesting not only due to its effects on regulating the flow of mitochondrial oxidative pathways but also given its role in controlling the production of reactive oxygen species^35^. Through this latter action, *SIRT3* activates enzymes responsible for quenching reactive oxygen species and thereby exerts a protective action against cellular oxidative stress. Thus, up-regulation of *SIRT3* in the visceral adipose of the HI calves may offer a protective effect from cellular reactive oxygen species and consequent cellular damage. Network 12 (Fig. 3) establishes a further link between differentially expressed genes involved in metabolism (*ABAT, GK, LDHB, LDHC, LDHD*), mitochondrial energy production and mitochondrial regulation (*COX20, FOXRED1, MT-ATP6, MT-CYB, MT-ND2, MT-ND3, MT-ND4L, PRDX3, SDHC, TXNRD2, UQCR10, UQCR11, UQCRC1, UQCRFS1*) and *SIRT3*. Similarly, genes involved in mitochondrial function were also differentially expressed in the subcutaneous adipose data of English et al.^28^ (*MT-ATP6, MT-ND2, MT-ND3, MT-ND4L, UQCR10, UQCR11, UQCRC1*). However, functional mitochondrial assays are required to be undertaken to fully establish the effect of enhanced dietary intake on visceral adipose mitochondrial capacity or efficiency. Additional genes differentially expressed between HI and MOD groups and also included within this network included *S100A10*, *S100B* and *SULF1*, all of which were consistently up-regulated in the adipose tissue of the HI calves compared to the MOD calves. Both *S100A10* and *S100B* code for S100 calcium binding proteins, which are involved in the regulation of cellular processes such as cell cycle progression and differentiation^36^. *SULF1* codes for an extracellular heparin sulphate endosulfatase, and may be involved in the regulation of activities of heparin sulphate binding growth factors including fibroblast growth factors and bone morphogenetic proteins^37^, both of which may impact adipose cell fate determination as well as regulating adipocyte function. Altogether, these results indicate an up-regulation of metabolism and oxidative phosphorylation processes as a consequence of enhanced early life nutrition, with *SIRT3* expression potentially providing a protective role against cellular damage with the increase in metabolic and oxidative processes in the HI group. Additionally within Network 12 it can be seen that *ERK* is the central gene within the network, with all other genes or processes included directed towards *ERK*. Although not differentially expressed within the current study, the greater expression of upstream genes of *ERK* within the HI group suggests that *ERK* expression may potentially be different if the altered plane of nutrition was sustained for a longer period. *ERK* forms part of the MAPK/ERK signalling pathway which is largely involved in cell proliferation, differentiation, transcription regulation and development^38^. Thus the genes included within this network may contribute to greater growth in visceral adipose through ERK/MAPK signalling in the HI group compared to the MOD group, as a direct consequence of increased metabolic processes.

### Adipogenesis

An increase in adipogenesis as a consequence of increased dietary intake has previously been reported by English et al.^28^ as well as by others^3^, whereby more pre-adipocytes may develop into a greater number of mature adipocytes as a consequence of greater dietary intake during the post-natal period. Thus the identification of the adipogenesis biochemical pathway as significantly enriched between the HI and MOD groups in the current study may have been expected. Throughout the body, adipocytes have a vital role in energy homeostasis and possess the largest energy reserve as triglycerol in the body^39^. Indeed, the energy reserve function of adipose tissue may be largely influenced by anabolic hormones of the endocrine system such as insulin and IGF-1, which have the capacity to contribute to adipogenesis, through inducing adipogenesis in pre-adipocytes^39^. Systemic concentrations of both IGF-1 and insulin of the heifers used in the current study were consistently higher in the HI calves throughout the trial^26^, thus there is potential that these two anabolic hormones may be contributing to the development of visceral adipose tissue and also to the differential expression of adipogenic genes. A number of genes that were differentially expressed in the current study are involved in the pre-adipocyte stage of differentiation, these included *AKT1*, *FGF1*, *FGFR3*, *PPIP5K1*. Both *AKT1* and *PPIP5K1* are involved in insulin mediated adipogenesis^40,41^, whereas *FGF1* and *FGFR3* are part of the fibroblast growth factor signalling^42^. The fold change direction of these genes, however, did not indicate an up-regulation of pre-adipocyte differentiation in the calves fed the high compared to the moderate plane of nutrition. *AKT1* and *PPIP5K1* were down and up-regulated, respectively in the high-nutrition compared to the moderate diet group. *AKT* is a critical kinase in the insulin-signalling cascade that is required for the process of adipogenesis^43^. Moreover Akt isoforms have been shown through gene knock-out studies to be involved in the determination of fat mass through the interaction with pre-adipocyte to adipocyte transition and regulating lipid storage^43^. Down-regulation of *AKT1* in the current study may have been manifested as a consequence of the up-regulation of *IP6K* (also known as *PPIP5K1)*, which functions to inhibit Akt signalling^44^. In a complementary study from our group, *PPIP5K1 (IP6K)* gene expression was also up-regulated in the subcutaneous adipose tissue of bull calves fed a high- compared to a moderate plane of nutrition^28^. Together these results suggest that any difference in adipogenesis in the current study may not be derived from insulin-mediated adipogenesis mechanisms.

*FGF1* which was up-regulated in the high calves compared to the moderate dietary group is involved in the fibroblast growth factor signalling pathway. This gene has also been shown to be up-regulated in adipose tissue in response to high-fat diets^45^. Furthermore, another member of the fibroblast growth factor family, *FGF2,* was up-regulated in bull calves offered a high compared to a moderate diet during the early life period^28^. However, in the current study, although *FGF1* was up-regulated, a receptor for this protein (*FGFR3*) was in fact down-regulated in the calves on the high plane of nutrition. Similarly the *KLF5* gene was down-regulated in the calves that were on the high diet. This gene has been shown to be induced in the early stages of differentiation of pre-adipocyte cell-lines^46^, suggesting that there was less pre-adipocyte differentiation at 21 weeks of age in the calves on the high plane of nutrition and that the calves on the HI diet were further along the overall adipogenesis trajectory. Overall though, there did not appear to be a clear effect of plane of nutrition on differential expression of pre-adipocyte genes.

While plane of nutrition did not induce a direct effect on the expression of pre-adipocyte related genes, those coding for proteins involved in the formation of mature adipocytes were up-regulated in HI calves. For example expression of *ARNTL* and *GTF2H5* were greater in the high-energy calves compared to their moderately-fed counterparts. The *ARNTL* gene modulates the regulation of adipocyte differentiation and lipogenesis^47^, whereas *GTH2H5* encodes a general transcription factor that has been shown to interact with adipocytes and be enriched in adipocyte precursor cells. Additionally, reduced expression of *PER2* and *TXNIP* genes may have contributed to increased adipogenesis. Specifically *PER2* functions to repress PPARg receptor activity which is critical in adipogenesis^48^, indicating greater adipogenesis overall in the current study. Similarly *TXNIP* expression was also lower in the high-energy calves, with deletion of this gene previously shown to cause adiposity and adipogenesis^49^. Finally, *AGPAT2* and *LEP* genes were both up-regulated in the calves on the high plane of nutrition compared to those on the moderate plane of nutrition, indicating a greater degree of adipogenesis in the high compared to the moderate group. This was manifested through the function of the *AGPAT2* gene which is critical for adipocyte growth and differentiation, producing both glycerophospholipids and triacylglycerols, with the latter being stored in adipocytes for subsequent conversion to energy, when required^50^. Additionally, *LEP* encodes a peptide hormone primarily produced in adipose tissue which conveys the biological energy status to the brain. Both *AGPAT2* and *LEP* were previously identified as up-regulated in bull calves fed a high-energy diet for the first 18 weeks of life^28^. Indeed, systemic concentrations of leptin were significantly higher in the HI calves compared to the MOD calves used for the current study at 20 weeks of age^26^. Together these results indicate that HI calves were further along the adipogenic trajectory compared to MOD calves following differential feeding up to 21 weeks of age. This effect is further established through results from our contemporary study in bull calves^28^, whereby early life nutrition was found to affect subcutaneous adipocyte number and size. More specifically, in that study bull calves were offered a dietary regime very similar to that employed for the current study and were fed moderate or enhanced diets for the first 18 weeks of life. Histological analyses of subcutaneous adipose at 18 weeks of age showed that bull calves on the High diet had greater adipocyte number and cell diameter when compared to their moderately fed counterparts^28^. However the current results also indicate that the heifer calves on the lower plane of nutrition had not yet completed pre-adipocyte differentiation at 21 weeks of age having been offered a typical moderate-energy diet. Cumulatively, our gene expression data show that offering a high plane of nutrition was associated with greater adipogenesis. However additional evaluations including for example histological and functional analyses of visceral adipose are required to prove this theory. This increase in adipogenesis may beneficially impact the lifelong growth and production potential of an animal including enhanced lactogenesis potential, earlier reproductive development and improved carcass composition.

### Reproductive development

As mentioned above the calves offered the high plane of nutrition displayed increased expression of the leptin gene in the visceral adipose. This peptide hormone is reflective of the body’s energy stores, conveying subsequent neuroendocrine signalling messages to the brain. For example, leptin signalling to the hypothalamus can subsequently convey a signal of fullness, leading to suppression of appetite^51^. However in addition to conveying a signal for satiety, leptin production in the adipose tissue and subsequent signalling to the brain can also lead to additional physiological functions for example, reproductive development^52^. Specifically, leptin can influence reproductive function, through a direct stimulatory effect on the hypothalamic-pituitary–gonadal (HPG) axis by accelerating gonadatropin-releasing hormone (GnRH) secretion in the hypothalamus^53,54^. Through the HPG axis, secretion of GnRH leads, in turn, to synthesis and secretion of the gonadotropins, FSH (follicle stimulating hormone) and LH (luteinizing hormone) from the anterior pituitary, which then signal to their target receptors located in the ovary, in females. Gonadotropin receptor binding in the ovary, leads to the production of estradiol (E2) and further reproductive development. Additionally, leptin can also have a direct effect on the anterior pituitary gland, directly stimulating the release of luteinizing hormone^55^. Thus, an increase in *LEP* gene expression as well as systemic concentrations of leptin^26^ may represent an early signal for the initiation of puberty and development of reproductive function^55^. This potential early development of reproductive development in the calves on the high plane of nutrition is further evidenced by the differential expression of genes directly involved in reproductive development and function. For example the *ESR1* gene which codes for estrogen receptor was up-regulated in the adipose tissue of the HI calves. The greater expression of this receptor within this dietary group is consistent with the higher systemic concentrations of E2 measured in the HI compared to the MOD calves^26^, implying a direct link between early life nutrition in heifer calves and subsequent steroidogenic capacity and reproductive development. Additionally, *GPER1* and *GREB1* which both code for estrogen responsive genes within the estrogen regulated signalling pathway^56,57^ were also up-regulated in the adipose tissue of the HI calves. Moreover, pathway analysis of up-regulated genes alone resulted in the enrichment of the estrogen receptor signalling pathway, indicating a potential activation of this pathway within the adipose tissue of the HI calves. Within the adipose tissue, E2 can elicit a number of downstream effects including cellular proliferation, adipose tissue distribution and glucose homeostasis^58,59^. Indeed the effects of *ESR1* in adipose tissue in the current study are highlighted within Network 2 (Fig. 2), where effector genes of ESR1 included those involved in processes such as metabolism (*ACSF2, ECH1, GMPPB, MPC2, NIT1, SORD*), cellular transport (*CLIC6, SLC25A20, SLC4A10, SLC4A11, SLC4A4, SLC4A5, XK*) and gene expression (*HSF2BP, MTERF2, RAD51B*). Thus the greater dietary intake of the HI calves may lead to earlier secretion of gonadotropins, with the E2 subsequently produced potentially contributing to adipogenesis. This theory is further strengthened by an evaluation of the development of the reproductive tract at 21 weeks of age, where the weight of the reproductive tract, number of surface follicles and number of oocytes were all greater in the HI calves compared to the MOD calves^26^.

In addition to an up-regulation of genes involved in estrogen receptor signalling, differential expression of genes involved in the biosynthesis of estrogen was also apparent in the adipose tissue of the HI calves. As well as being a target tissue for hormone receptors, adipose tissue represents an important endocrine organ within the body and produces various hormones including estrogen^60^. *HSD17B12*, which codes for estradiol 17-beta dehydrogenase 12, was up-regulated in the calves on the high diet compared to the low diet. *HSD17B12* codes for an enzyme that catalyses the transformation of estrone into estradiol, ultimately contributing to estrogen formation^61^. Furthermore, *SRD5A1* which encodes an enzyme responsible for the final step of androsterone production and is also a precursor to estrogen production^62^ also displayed greater adipose expression in the calves on the high plane of nutrition compared to the moderate plane of nutrition. *SRD5A1* was also up-regulated in the subcutaneous adipose of bull calves that were fed an enhanced early life energy diet^28^. Both *SRD5A1* and *HSD17B12* are steroidogenic genes and were both up-regulated in the high compared to the moderate energy intake group. These differential gene expression results indicate that the calves consuming the higher plane of nutrition may have had the potential to produce estradiol within the adipose tissue. Typically E2 production is most associated with being an endocrine product of the ovary, however there are many extraglandular tissues including adipose that are capable of synthesizing estrogens^60^. This is achieved through the enzyme aromatase catalysing the conversion of C(19) steroids to estrogens^63^. Adipose tissue in particular can contribute significantly to the circulating pool of estrogens^64^, with the amount produced rising with increased bodyweight and age. Overall, these results indicate that the adipose tissue of the calves on the HI diet may be contributing to the earlier development of the reproductive tract through either greater gene expression and systemic production of leptin, subsequently leading to earlier gonadotropin release or directly through E2 production. Earlier reproductive development and more precocious attainment of puberty are undoubtedly beneficial for the efficient recruitment and retention of replacement heifers in both beef and dairy cattle herds. However, further analyses are warranted in order to fully elucidate the effect of enhanced early life nutrition toward adipose derived E2 production, through for example measurement of adipose tissue E2 concentrations.

## Conclusions

Results from this study clearly show an effect of early life plane of nutrition on the molecular control of the visceral adipose transcriptome in heifer calves. Calves offered an enhanced energy diet for the first 21 weeks of life displayed greater expression of genes involved in metabolic processes including lipid and amino acid metabolism as well as greater expression of mitochondrial oxidative phosphorylation genes. Additionally, results suggest a key role for *SIRT3* to the greater metabolic and mitochondrial functions association with the enhanced diet of the HI group. Adipogenesis genes differentially expressed as a result of differential feeding for the first 21 weeks of life suggested that the adipose tissue of the HI calves was further along the overall adipogenesis trajectory, with greater expression of genes involved in the formation of mature adipocytes apparent in HI calves. Finally the greater expression of *LEP* in HI calves may have contributed to the earlier reproductive development in these calves as a direct consequence of dietary intake through augmenting the HPG signalling axis. Additionally there is evidence to suggest that the higher plane of nutrition may also contribute to increased E2 synthesis in visceral adipose tissue. Overall the transcriptional profiling results from this study indicate an increase in adipogenesis and potential contribution to reproductive development, both of which may impact the lifelong growth and production potential of an animal including enhanced lactogenesis potential, earlier reproductive development and improved carcass composition.

## Supplementary Information

Supplementary Information

## Data Availability

The dataset analysed during the current study is available in the NCBI Gene Expression Omnibus https://www.ncbi.nlm.nih.gov/geo/ under Accession Number GSE158128.
