# Supplementary material for: Effect of plane of nutrition in early life on the transcriptome of visceral adipose tissue in Angus heifer calves
Source: Sci Rep. 2021 May 6;11:9716. doi: 10.1038/s41598-021-89252-x (PMC8102595; doi:10.1038/s41598-021-89252-x)
Supplement: Supplementary file 1 — Supplementary Information [file 41598_2021_89252_MOESM1_ESM.pdf]

Supplementary information

Effect of plane of nutrition in early life on the transcriptome of visceral adipose tissue in Angus heifer calves

Authors: Kate Keogh[1], Alan K. Kelly[2] and David A. Kenny[1,2*].
[1]Teagasc Animal & Bioscience Research Department, Teagasc Grange, Dunsany, Co Meath, Ireland.

[2]School of Agriculture and Food Science, University College Dublin, Belfield, Dublin 4, Ireland.

*Corresponding Author: Prof. David Kenny;

email: david.kenny@teagasc.ie

Supplementary Table 1: Genes identified as differentially expressed and mapped in Ingenuity Pathway Analysis (IPA) in visceral adipose of heifer calves offered either a High plane of nutrition or a Moderate plane of nutrition for the first 21 weeks of life.

Symbol[1]	Entrez Gene Name	Fold Change[2]	q-value
2410002F23Rik	RIKEN cDNA 2410002F23 gene	-1.518	0.0227
ABAT	4-aminobutyrate aminotransferase	2.063	0.0104
ABCA7	ATP binding cassette subfamily A member 7	3.14	3.5E-16
ABCB6	ATP binding cassette subfamily B member 6 (Langereis blood group)	1.641	0.00245
ABCC8	ATP binding cassette subfamily C member 8	2.23	0.000281
ABCD1	ATP binding cassette subfamily D member 1	1.756	0.000199
ABHD12	Abhydrolase domain containing 12	1.551	0.00742
ACADS	Acyl-CoA dehydrogenase short chain	1.909	0.000404
ACADSB	Acyl-CoA dehydrogenase short/branched chain	1.647	0.00687
ACAT2	Acetyl-CoA acetyltransferase 2	2.149	0.000933
ACBD4	Acyl-CoA binding domain containing 4	1.767	0.0249
ACE2	Angiotensin I converting enzyme 2	2.862	0.0000325
ACKR3	Atypical chemokine receptor 3	1.625	0.00255
ACLY	ATP citrate lyase	4.067	0.000000536
Acot6	Acyl-CoA thioesterase 6	2.521	0.00281
ACOX1	Acyl-CoA oxidase 1	1.547	0.0423
ACOX2	Acyl-CoA oxidase 2	1.811	0.0458
ACSBG1	Acyl-CoA synthetase bubblegum family member 1	1.645	0.000511
ACSF2	Acyl-CoA synthetase family member 2	1.628	0.0264
ACSM2A	Acyl-CoA synthetase medium chain family member 2A	-2.585	0.0499
ACSM3	Acyl-CoA synthetase medium chain family member 3	6.262	0.00888
ACSS2	Acyl-CoA synthetase short chain family member 2	3.041	0.00000598
ACYP2	Acylphosphatase 2	1.955	0.0000206
ADAM11	ADAM metallopeptidase domain 11	-2.454	0.00634
ADAM19	ADAM metallopeptidase domain 19	1.584	0.0011
ADAM9	ADAM metallopeptidase domain 9	1.605	0.0205
ADAMTS16	ADAM metallopeptidase with thrombospondin type 1 motif 16	2.708	0.00146
ADAMTS17	ADAM metallopeptidase with thrombospondin type 1 motif 17	2.222	0.0000048
ADAMTS2	ADAM metallopeptidase with thrombospondin type 1 motif 2	1.529	0.000777
ADH6	Alcohol dehydrogenase 6 (class V)	3.083	0.00000329
ADHFE1	Alcohol dehydrogenase, iron containing 1	2.463	0.00000285

ADTRP	Androgen dependent TFPI regulating protein	1.516	0.0256
AES	Amino-terminal enhancer of split	-1.561	0.0011
AGPAT2	1-acylglycerol-3-phosphate O-acyltransferase 2	1.652	0.0139
AIFM3	Apoptosis inducing factor, mitochondria associated 3	-2.592	0.000236
AK2	Adenylate kinase 2	-1.513	0.0124
AK5	Adenylate kinase 5	-1.832	0.0195
AKAP12	A-kinase anchoring protein 12	1.668	0.00731
AKAP4	A-kinase anchoring protein 4	-4.814	0.000337
AKR1C3	Aldo-keto reductase family 1 member C3	-2.092	0.00144
AKR7A2	Aldo-keto reductase family 7 member A2	1.748	0.00503
AKT1	AKT serine/threonine kinase 1	-1.515	0.000398
ALDH18A1	Aldehyde dehydrogenase 18 family member A1	1.532	0.043
ALDH2	Aldehyde dehydrogenase 2 family member	1.635	0.00017
ALDH4A1	Aldehyde dehydrogenase 4 family member A1	1.747	0.000333
ALDOA	Aldolase, fructose-bisphosphate A	1.755	0.00139
ALDOC	Aldolase, fructose-bisphosphate C	2.548	0.000000551
AMIGO3	Adhesion molecule with Ig like domain 3	-1.607	0.014
ANGPT2	Angiopoietin 2	-1.806	0.0042
ANGPTL8	Angiopoietin like 8	7.831	0.000032
ANKRD35	Ankyrin repeat domain 35	2.796	2.02E-08
ANKRD6	Ankyrin repeat domain 6	-1.816	0.00066
ANP32A	Acidic nuclear phosphoprotein 32 family member A	-1.501	0.02
ANP32B	Acidic nuclear phosphoprotein 32 family member B	-1.501	0.00981
AOC2	Amine oxidase, copper containing 2	1.796	0.0002
AP3B2	Adaptor related protein complex 3 subunit beta 2	-5.342	2.53E-12
APCDD1	APC down-regulated 1	-4.299	0.000000611
APOBEC2	Apolipoprotein B mRNA editing enzyme catalytic subunit 2	1.693	0.0105
APOL2	Apolipoprotein L2	2.031	0.00182
AQP3	Aquaporin 3 (Gill blood group)	-2.219	0.001
ARHGAP22	Rho GTPase activating protein 22	1.929	0.00127
ARHGEF19	Rho guanine nucleotide exchange factor 19	1.779	0.00688
ARHGEF37	Rho guanine nucleotide exchange factor 37	1.78	0.00581
ARID3A	AT-rich interaction domain 3A	-2.183	0.0000193
ARMC12	Armadillo repeat containing 12	-2.181	0.00944
ARNTL	Aryl hydrocarbon receptor nuclear translocator like	1.586	0.0172

ARRDC2	Arrestin domain containing 2	-1.775	0.0131
ASB16	Ankyrin repeat and SOCS box containing 16	-2.13	0.00066
ASB2	Ankyrin repeat and SOCS box containing 2	4.316	5.19E-16
ASB9	Ankyrin repeat and SOCS box containing 9	1.825	0.00742
ASGR1	Asialoglycoprotein receptor 1	-2.537	0.0000192
ASGR2	Asialoglycoprotein receptor 2	1.711	0.0345
ASL	Argininosuccinate lyase	-1.817	0.0000472
ASMTL	Acetylserotonin O-methyltransferase like	1.583	0.0217
ASS1	Argininosuccinate synthase 1	-1.722	0.00139
ASZ1	Ankyrin repeat, SAM and basic leucine zipper domain containing 1	2.177	0.00938
ATL2	Atlastin GTPase 2	2.022	0.00212
ATOX1	Antioxidant 1 copper chaperone	1.953	0.000915
ATP10A	ATPase phospholipid transporting 10A (putative)	-1.768	0.00435
ATP13A4	ATPase 13A4	-2.406	0.00105
ATP2B2	ATPase plasma membrane Ca2+ transporting 2	1.943	0.00413
ATP5MF	ATP synthase membrane subunit f	1.884	0.0114
ATP6V1E1	ATPase H+ transporting V1 subunit E1	1.714	0.000404
ATRNL1	Attractin like 1	1.763	0.0164
AVPI1	Arginine vasopressin induced 1	-2.425	0.000644
B3GAT1	Beta-1,3-glucuronyltransferase 1	-2.034	0.000573
BAIAP2L1	BAI1 associated protein 2 like 1	2.657	0.0000016
BANP	BTG3 associated nuclear protein	-1.703	0.00000285
BCAP31	B cell receptor associated protein 31	1.739	0.00128
BCL6	B cell CLL/lymphoma 6	2.501	0.000000115
BDH1	3-hydroxybutyrate dehydrogenase 1	6.348	0.0000375
BICD1	BICD cargo adaptor 1	2.082	0.000903
BIN3	Bridging integrator 3	-1.753	0.000199
BMPER	BMP binding endothelial regulator	2.876	0.000557
BNIP3	BCL2 interacting protein 3	-2.019	0.000903
BOLA3	BolA family member 3	2.274	0.00462
BPHL	Biphenyl hydrolase like	1.615	0.000607
BUD23	BUD23, rRNA methyltransferase and ribosome maturation factor	1.598	0.0328
C1QTNF2	C1q and TNF related 2	1.691	0.00734
C2	Complement C2	-1.902	0.00209
C2CD2	C2 calcium dependent domain containing 2	-1.607	0.0128

C3orf18	Chromosome 3 open reading frame 18	1.769	0.00176
C9orf50	Chromosome 9 open reading frame 50	1.824	0.0155
CA10	Carbonic anhydrase 10	2.452	0.0103
CA11	Carbonic anhydrase 11	2.859	0.000188
CA4	Carbonic anhydrase 4	-1.511	0.0499
CABCOCO1	Ciliary associated calcium binding coiled-coil 1	-2.702	0.0000298
CACNA1G	Calcium voltage-gated channel subunit alpha1 G	1.581	0.00834
CALR3	Calreticulin 3	2.278	0.0153
CAMK1G	Calcium/calmodulin dependent protein kinase IG	-5.403	0.0000207
CAMP	Cathelicidin antimicrobial peptide	5.91	0.037
CAPN3	Calpain 3	2.408	0.000337
CAST	Calpastatin	-1.505	0.00335
CCDC112	Coiled-coil domain containing 112	-1.566	0.0071
CCDC115	Coiled-coil domain containing 115	1.554	0.00887
CCDC3	Coiled-coil domain containing 3	3.53	2.95E-09
CCDC69	Coiled-coil domain containing 69	1.831	0.00496
CCDC8	Coiled-coil domain containing 8	-1.596	0.00163
CCDC85C	Coiled-coil domain containing 85C	2.296	0.000000783
CCDC92	Coiled-coil domain containing 92	1.562	0.00371
CCND2	Cyclin D2	-1.956	0.000167
CCNG2	Cyclin G2	2.017	7.88E-09
CD1A	CD1a molecule	1.897	0.00097
CD81	CD81 molecule	1.5	0.0227
CDC25A	Cell division cycle 25A	1.63	0.0499
CDC34	Cell division cycle 34	1.876	0.00293
CDC42EP4	CDC42 effector protein 4	-1.636	0.00232
CDK18	Cyclin dependent kinase 18	-1.73	1.54E-08
CDS1	CDP-diacylglycerol synthase 1	2.482	0.0114
CENPX	Centromere protein X	1.656	0.017
CEP89	Centrosomal protein 89	-1.538	0.00139
CERS6	Ceramide synthase 6	1.807	0.00921
CFI	Complement factor I	1.59	0.0358
CGNL1	Cingulin like 1	1.875	0.00487
CGREF1	Cell growth regulator with EF-hand domain 1	1.577	0.0154
CHAC2	ChaC cation transport regulator homolog 2	1.637	0.0038

CHAF1A	Chromatin assembly factor 1 subunit A	2.24	0.00000216
CHI3L1	Chitinase 3 like 1	2.373	0.0000213
CHPT1	Choline phosphotransferase 1	1.663	0.000173
CHRNA3	Cholinergic receptor nicotinic alpha 3 subunit	-2.207	0.00458
CIB4	Calcium and integrin binding family member 4	-1.761	0.0387
CILP2	Cartilage intermediate layer protein 2	-1.501	0.0341
CIRBP	Cold inducible RNA binding protein	-1.641	0.00208
CISD1	CDGSH iron sulfur domain 1	2.305	0.000156
CISH	Cytokine inducible SH2 containing protein	-2.019	0.00472
CKB	Creatine kinase B	4.6	0.00000187
CLCF1	Cardiotrophin like cytokine factor 1	-1.815	0.000398
CLCN4	Chloride voltage-gated channel 4	2.039	0.00503
Clec2g	C-type lectin domain family 2, member G	-2.95	1.82E-24
CLGN	Calmegin	1.866	0.0141
CLIC6	Chloride intracellular channel 6	-2.715	0.00896
CLMN	Calmin	-1.609	0.0013
CLU	Clusterin	3.24	1.46E-08
CMC2	C-X9-C motif containing 2	2.247	0.000337
CNKSR3	CNKSR family member 3	-1.502	0.0334
CNTN1	Contactin 1	1.531	0.0122
COA3	Cytochrome c oxidase assembly factor 3	1.857	0.00148
COA7	Cytochrome c oxidase assembly factor 7 (putative)	1.932	0.000288
COL11A2	Collagen type XI alpha 2 chain	-2.17	0.000333
COL1A1	Collagen type I alpha 1 chain	1.508	0.025
COL1A2	Collagen type I alpha 2 chain	1.662	0.00412
COL20A1	Collagen type XX alpha 1 chain	-3.604	0.000599
COL22A1	Collagen type XXII alpha 1 chain	-6.502	0.00000224
COL5A3	Collagen type V alpha 3 chain	1.825	0.00000637
COPS9	COP9 signalosome subunit 9	1.549	0.0381
COQ9	Coenzyme Q9	1.543	0.0227
CORIN	Corin, serine peptidase	-10.078	9.97E-13
CORO2A	Coronin 2A	-2.611	0.000000684
COX20	Cytochrome c oxidase assembly factor COX20	1.658	0.0477
COX4I2	Cytochrome c oxidase subunit 4I2	1.753	0.0205
Cox6c	Cytochrome c oxidase subunit 6C	1.967	0.00623

COX7A1	Cytochrome c oxidase subunit 7A1	2.512	0.00139
COX7B	Cytochrome c oxidase subunit 7B	1.627	0.0251
CPAMD8	C3 and PZP like, alpha-2-macroglobulin domain containing 8	-1.774	0.0114
CPE	Carboxypeptidase E	1.719	0.0349
CPNE2	Copine 2	-1.628	0.000778
CPT2	Carnitine palmitoyltransferase 2	1.662	0.00192
CPXM1	Carboxypeptidase X, M14 family member 1	1.679	0.000238
CPZ	Carboxypeptidase Z	2.177	0.00435
CRABP2	Cellular retinoic acid binding protein 2	1.848	0.0476
CRAT	Carnitine O-acetyltransferase	1.777	0.00813
CRB1	Crumbs 1, cell polarity complex component	1.883	0.00906
CRYAB	Crystallin alpha B	2.75	0.00188
CRYZ	Crystallin zeta	2.053	0.00000774
CSDC2	Cold shock domain containing C2	2.278	0.0019
CSPG4	Chondroitin sulfate proteoglycan 4	-1.546	0.00301
CSRNP1	Cysteine and serine rich nuclear protein 1	-1.533	0.00694
CTSD	Cathepsin D	1.669	0.00051
CTSO	Cathepsin O	-1.638	0.0132
CX3CL1	C-X3-C motif chemokine ligand 1	1.575	0.00728
CX3CR1	C-X3-C motif chemokine receptor 1	1.577	0.0447
CXCL2	C-X-C motif chemokine ligand 2	-2.215	0.00621
CYB5A	Cytochrome b5 type A	1.927	0.000183
CYP4V2	Cytochrome P450 family 4 subfamily V member 2	-2.562	0.0000346
DAPK3	Death associated protein kinase 3	-1.655	0.00196
DBI	Diazepam binding inhibitor, acyl-CoA binding protein	1.855	0.0195
DBP	D-box binding PAR bZIP transcription factor	-1.57	0.0363
DCDC2B	Doublecortin domain containing 2B	-1.717	0.00748
DCLRE1B	DNA cross-link repair 1B	1.969	0.0406
DDAH2	Dimethylarginine dimethylaminohydrolase 2	-2.12	0.00277
DDHD2	DDHD domain containing 2	1.614	0.000282
DDIT4	DNA damage inducible transcript 4	-2.401	0.000224
DECR1	2,4-dienoyl-CoA reductase 1	2.355	0.000228
DENND2D	DENN domain containing 2D	-2.121	0.00000275
DEPP1	DEPP1, autophagy regulator	-3.286	0.000165
DGAT2	Diacylglycerol O-acyltransferase 2	1.802	0.0459

DHCR24	24-dehydrocholesterol reductase	2.014	0.00851
DHRS3	Dehydrogenase/reductase 3	1.714	0.0000145
DKK3	Dickkopf WNT signaling pathway inhibitor 3	-1.647	0.0342
DLX5	Distal-less homeobox 5	1.763	0.0175
DMPK	DM1 protein kinase	2.637	0.000569
DMTN	Dematin actin binding protein	-2.699	0.00000594
DNAJB5	DnaJ heat shock protein family (Hsp40) member B5	1.623	0.0223
DNAJC12	DnaJ heat shock protein family (Hsp40) member C12	1.742	0.0098
DNAJC24	DnaJ heat shock protein family (Hsp40) member C24	1.529	0.00914
DNAJC6	DnaJ heat shock protein family (Hsp40) member C6	-2.064	0.000777
DNAL4	Dynein axonemal light chain 4	-1.508	0.0388
DNHD1	Dynein heavy chain domain 1	-1.745	0.00821
DOCK8	Dedicator of cytokinesis 8	-2.247	0.0000634
DPYSL3	Dihydropyrimidinase like 3	-2.193	0.000332
DQX1	DEAQ-box RNA dependent ATPase 1	-1.711	0.000753
DUSP10	Dual specificity phosphatase 10	3.057	1.05E-18
DUSP22	Dual specificity phosphatase 22	-1.769	0.0000173
DUSP26	Dual specificity phosphatase 26	-6.226	0.0000089
DUSP4	Dual specificity phosphatase 4	-2.386	0.011
DYNLRB2	Dynein light chain roadblock-type 2	-2.505	0.0141
ECH1	Enoyl-CoA hydratase 1	1.556	0.00677
ECHDC2	Enoyl-CoA hydratase domain containing 2	1.91	0.00000175
ECHS1	Enoyl-CoA hydratase, short chain 1	1.723	0.00504
EEF2K	Eukaryotic elongation factor 2 kinase	-1.689	0.00326
EFS	Embryonal Fyn-associated substrate	-1.771	1.62E-08
EHBP1	EH domain binding protein 1	-1.652	0.00688
EIF2B2	Eukaryotic translation initiation factor 2B subunit beta	1.516	0.00853
EIF4G1	Eukaryotic translation initiation factor 4 gamma 1	1.529	0.00308
ELL3	Elongation factor for RNA polymerase II 3	-1.606	0.0235
ELOVL1	ELOVL fatty acid elongase 1	-1.501	0.00066
ELOVL5	ELOVL fatty acid elongase 5	1.938	0.00947
ELP6	Elongator acetyltransferase complex subunit 6	1.755	0.00267
EMILIN2	Elastin microfibril interfacer 2	-1.681	0.0167
ENO4	Enolase 4	-1.754	0.0182
ENPEP	Glutamyl aminopeptidase	2.224	0.0000321

ENTPD2	Ectonucleoside triphosphate diphosphohydrolase 2	1.843	0.00136
EPB41L4B	Erythrocyte membrane protein band 4.1 like 4B	-1.76	0.0287
EPDR1	Ependymin related 1	1.782	0.0083
EPHX2	Epoxide hydrolase 2	1.985	0.00000182
EPM2A	EPM2A, laforin glucan phosphatase	1.614	0.0147
ERBB2	Erb-b2 receptor tyrosine kinase 2	-1.933	0.0000355
ESR1	Estrogen receptor 1	2.199	0.0109
ETFA	Electron transfer flavoprotein subunit alpha	2.022	0.00162
ETFDH	Electron transfer flavoprotein dehydrogenase	1.603	0.0151
ETV5	ETS variant 5	-1.536	0.0442
F13A1	Coagulation factor XIII A chain	1.517	0.0257
FAAP100	FA core complex associated protein 100	-1.608	0.00103
FAM155B	Family with sequence similarity 155 member B	2.054	0.00941
FAM19A5	Family with sequence similarity 19 member A5, C-C motif chemokine like	-2.028	0.00326
FAM210B	Family with sequence similarity 210 member B	-2.162	0.00401
FAM221B	Family with sequence similarity 221 member B	-2.639	0.000075
FAM84A	Family with sequence similarity 84 member A	1.939	0.000535
FANCD2	FA complementation group D2	2.021	0.00182
FAP	Fibroblast activation protein alpha	1.649	0.0152
FASN	Fatty acid synthase	2.711	0.0159
FAT1	FAT atypical cadherin 1	-1.77	0.00479
FBP1	Fructose-bisphosphatase 1	-2.565	2.84E-11
FBXO17	F-box protein 17	-2.121	0.0000168
FBXO4	F-box protein 4	-1.588	0.000572
FBXO40	F-box protein 40	1.951	0.00106
FCN1	Ficolin 1	-2.04	0.000699
FFAR4	Free fatty acid receptor 4	2.175	0.000422
FGF1	Fibroblast growth factor 1	5.182	4.83E-15
FGFR3	Fibroblast growth factor receptor 3	-2.088	0.00267
FGL1	Fibrinogen like 1	1.704	0.0158
FH	Fumarate hydratase	1.632	0.00418
FHL1	Four and a half LIM domains 1	-3.535	1.77E-13
FKBP11	FK506 binding protein 11	-1.965	0.000182
FKRP	Fukutin related protein	-1.539	0.000231
FN3K	Fructosamine 3 kinase	1.738	0.0182

FNDC10	Fibronectin type III domain containing 10	1.767	0.000175
FNTB	Farnesyltransferase, CAAX box, beta	1.659	0.000364
Folh1	Folate hydrolase 1	2.638	3.31E-08
FOXP3	Forkhead box P3	1.839	0.00725
FOXRED1	FAD dependent oxidoreductase domain containing 1	1.625	0.00497
FPGS	Folylpolyglutamate synthase	-1.651	0.0000168
FUNDC2	FUN14 domain containing 2	1.596	0.000337
FUT1	Fucosyltransferase 1 (H blood group)	-1.75	0.000607
FXYD7	FXYD domain containing ion transport regulator 7	-1.806	0.0482
G0S2	G0/G1 switch 2	-2.304	0.00152
G6PD	Glucose-6-phosphate dehydrogenase	2.684	0.000000119
GALNT15	Polypeptide N-acetylgalactosaminyltransferase 15	-2.03	0.0264
GALNT16	Polypeptide N-acetylgalactosaminyltransferase 16	1.693	0.0000275
GAMT	Guanidinoacetate N-methyltransferase	1.587	0.0159
GAS2L2	Growth arrest specific 2 like 2	-2.465	0.0000652
GATM	Glycine amidinotransferase	-1.529	0.0273
GJC2	Gap junction protein gamma 2	1.75	0.00119
GK	Glycerol kinase	1.816	0.00274
GLRX5	Glutaredoxin 5	1.828	0.00596
GLT8D2	Glycosyltransferase 8 domain containing 2	1.533	0.000893
GLYAT	Glycine-N-acyltransferase	2.449	0.000736
GM2A	GM2 ganglioside activator	2.098	0.0000161
Gm4952	Predicted gene 4952	2.107	0.00114
GMEB2	Glucocorticoid modulatory element binding protein 2	-1.515	0.00136
GMPPB	GDP-mannose pyrophosphorylase B	-1.566	0.00519
GOT2	Glutamic-oxaloacetic transaminase 2	1.634	0.0182
GPAM	Glycerol-3-phosphate acyltransferase, mitochondrial	1.645	0.00496
GPC1	Glypican 1	1.616	0.0122
GPER1	G protein-coupled estrogen receptor 1	1.889	0.000357
GPI	Glucose-6-phosphate isomerase	1.969	0.0000318
Gpm6b	Glycoprotein m6b	1.613	0.0231
GPR156	G protein-coupled receptor 156	-1.838	0.00996
GPR21	G protein-coupled receptor 21	1.668	0.000903
GPR37	G protein-coupled receptor 37	1.843	0.0336
GPR37L1	G protein-coupled receptor 37 like 1	1.691	0.0259

GPRC5A	G protein-coupled receptor class C group 5 member A	-1.91	0.0265
GRB7	Growth factor receptor bound protein 7	-2.055	0.0489
GREB1	Growth regulating estrogen receptor binding 1	3.379	0.000068
GRIK2	Glutamate ionotropic receptor kainate type subunit 2	-2.199	0.000253
GSDMB	Gasdermin B	-2.055	0.00439
GSDME	Gasdermin E	-4.119	0.0000375
GSN	Gelsolin	-1.531	0.0175
GSS	Glutathione synthetase	1.707	0.0414
GSTA1	Glutathione S-transferase alpha 1	2.903	0.00115
GSTA4	Glutathione S-transferase alpha 4	1.67	0.0045
GSTM4	Glutathione S-transferase mu 4	1.877	0.0229
Gstt1	Glutathione S-transferase, theta 1	2.061	0.00302
Gstt3	Glutathione S-transferase, theta 3	2.273	0.0124
GTF2H5	General transcription factor IIH subunit 5	1.522	0.0214
GUCY2D	Guanylate cyclase 2D, retinal	2.622	0.000893
Gulo	Gulonolactone (L-) oxidase	1.685	0.0291
GYG2	Glycogenin 2	-1.545	0.0018
GYS2	Glycogen synthase 2	2.702	0.00686
HABP4	Hyaluronan binding protein 4	1.654	0.0141
HACD2	3-hydroxyacyl-CoA dehydratase 2	1.648	0.00000784
HADH	Hydroxyacyl-CoA dehydrogenase	1.514	0.00261
HAGHL	Hydroxyacylglutathione hydrolase like	1.827	0.000309
HAS2	Hyaluronan synthase 2	-9.507	0.000000562
HBD	Hemoglobin subunit delta	4.833	0.0147
HCAR3	Hydroxycarboxylic acid receptor 3	-1.659	0.00115
HCN2	Hyperpolarization activated cyclic nucleotide gated potassium and sodium channel 2	5.313	0.000000119
HCRTR1	Hypocretin receptor 1	-1.55	0.000906
HDC	Histidine decarboxylase	1.951	0.0156
HDGFL3	HDGF like 3	-1.957	0.000252
HES6	Hes family bHLH transcription factor 6	-5.31	0.0000293
HEY1	Hes related family bHLH transcription factor with YRPW motif 1	-1.85	0.000113
HIBADH	3-hydroxyisobutyrate dehydrogenase	1.509	0.000654
HIF1A	Hypoxia inducible factor 1 subunit alpha	-1.954	0.00512
HIF3A	Hypoxia inducible factor 3 subunit alpha	-2.542	0.000228
HIGD1B	HIG1 hypoxia inducible domain family member 1B	2.596	0.000174

HINT3	Histidine triad nucleotide binding protein 3	1.641	0.00503
HIST1H2AC	Histone cluster 1 H2A family member c	2.637	0.0000706
HIST1H2BN	Histone cluster 1 H2B family member n	1.996	0.00139
HIST1H2BO	Histone cluster 1 H2B family member o	1.516	0.000992
HIST1H4J	Histone cluster 1 H4 family member j	-1.762	0.0397
HIST2H4A	Histone cluster 2 H4 family member a	2.362	8.65E-09
HLA-DMB	Major histocompatibility complex, class II, DM beta	1.531	0.0476
HLCS	Holocarboxylase synthetase	1.795	0.000871
HMGB2	High mobility group box 2	-1.914	0.00127
HMOX2	Heme oxygenase 2	1.518	0.0158
HNRNPA1	Heterogeneous nuclear ribonucleoprotein A1	-1.719	0.00002
HOMER1	Homer scaffold protein 1	2.146	0.000281
HOXC5	Homeobox C5	1.805	0.00342
HOXC8	Homeobox C8	-1.876	0.0000652
HPD	4-hydroxyphenylpyruvate dioxygenase	3.949	0.00182
HSD17B11	Hydroxysteroid 17-beta dehydrogenase 11	-1.56	0.0097
HSD17B12	Hydroxysteroid 17-beta dehydrogenase 12	1.891	0.017
HSD17B14	Hydroxysteroid 17-beta dehydrogenase 14	2.512	0.000209
HSF2BP	Heat shock transcription factor 2 binding protein	-1.784	0.0038
HSPB6	Heat shock protein family B (small) member 6	1.625	0.00000257
IDH3B	Isocitrate dehydrogenase 3 (NAD(+)) beta	1.642	0.02
IDI1	Isopentenyl-diphosphate delta isomerase 1	2.051	0.0000813
IFIT3	Interferon induced protein with tetratricopeptide repeats 3	1.768	0.0272
IFT81	Intraflagellar transport 81	-1.505	0.0229
IGDCC4	Immunoglobulin superfamily DCC subclass member 4	1.904	0.0229
IGF1	Insulin like growth factor 1	-1.574	0.00693
IGFALS	Insulin like growth factor binding protein acid labile subunit	-2.524	0.0000297
IL1B	Interleukin 1 beta	1.631	0.0297
IL20RA	Interleukin 20 receptor subunit alpha	-5.039	2.82E-12
IL23A	Interleukin 23 subunit alpha	1.872	0.00011
IL2RA	Interleukin 2 receptor subunit alpha	2.995	3.31E-08
IL6R	Interleukin 6 receptor	-1.991	0.0138
IMPA2	Inositol monophosphatase 2	-1.683	0.00551
INPP5K	Inositol polyphosphate-5-phosphatase K	2.324	5.16E-11
INSR	Insulin receptor	-1.551	0.0351

IPO4	Importin 4	-1.803	0.000263
IRF8	Interferon regulatory factor 8	1.689	0.0101
IRS2	Insulin receptor substrate 2	-1.857	0.0158
IRX3	Iroquois homeobox 3	-1.59	0.0497
ISCU	Iron-sulfur cluster assembly enzyme	1.795	0.000457
ISOC1	Isochorismatase domain containing 1	1.586	0.00194
ISOC2	Isochorismatase domain containing 2	2.119	0.000804
ITGA7	Integrin subunit alpha 7	2.591	4.23E-09
ITGAE	Integrin subunit alpha E	1.631	0.00167
ITGBL1	Integrin subunit beta like 1	1.714	0.0259
ITIH3	Inter-alpha-trypsin inhibitor heavy chain 3	-2.834	0.000648
ITIH4	Inter-alpha-trypsin inhibitor heavy chain family member 4	-3.013	0.0000115
ITM2B	Integral membrane protein 2B	1.698	0.0000177
ITM2C	Integral membrane protein 2C	-1.528	0.0148
ITPKC	Inositol-trisphosphate 3-kinase C	-1.76	0.00124
IVD	Isovaleryl-CoA dehydrogenase	1.549	0.00537
IVNS1ABP	Influenza virus NS1A binding protein	-1.664	0.00198
KALRN	Kalirin RhoGEF kinase	-1.726	0.0011
KAZN	Kazrin, periplakin interacting protein	2.078	0.00058
KCNC3	Potassium voltage-gated channel subfamily C member 3	3.771	0.0000821
KCNC4	Potassium voltage-gated channel subfamily C member 4	-1.719	0.0104
KCNH3	Potassium voltage-gated channel subfamily H member 3	-3.782	0.0000465
KCNJ11	Potassium voltage-gated channel subfamily J member 11	2.745	0.00000863
KCNK17	Potassium two pore domain channel subfamily K member 17	-2.535	0.00049
KCNK2	Potassium two pore domain channel subfamily K member 2	2.212	0.00219
KCNMB1	Potassium calcium-activated channel subfamily M regulatory beta subunit 1	-1.67	0.0343
KCNMB4	Potassium calcium-activated channel subfamily M regulatory beta subunit 4	-1.75	0.00213
KCTD2	Potassium channel tetramerization domain containing 2	-1.554	0.000993
KDSR	3-ketodihydrosphingosine reductase	1.691	0.0000561
KHK	Ketohexokinase	2.253	0.000016
KIAA0408	KIAA0408	-1.929	0.00139
KIAA1614	KIAA1614	-1.795	0.000152
KIF1C	Kinesin family member 1C	1.599	0.000238
KIF24	Kinesin family member 24	1.946	0.0000821
KIF7	Kinesin family member 7	-1.728	0.00147

KIZ	Kizuna centrosomal protein	-1.713	0.00783
KLF11	Kruppel like factor 11	-1.609	0.0063
KLF4	Kruppel like factor 4	-1.844	0.00291
KLF5	Kruppel like factor 5	-2.21	0.00167
KLHDC8B	Kelch domain containing 8B	1.617	0.00834
KLHL25	Kelch like family member 25	-2.818	3.67E-08
KLHL33	Kelch like family member 33	1.698	0.01
KLK7	Kallikrein related peptidase 7	3.095	0.00146
KPNA2	Karyopherin subunit alpha 2	1.623	0.000658
LAMB1	Laminin subunit beta 1	-1.797	0.0000375
LAMC2	Lminin subunit gamma 2	2.093	0.0379
LAMTOR1	Late endosomal/lysosomal adaptor, MAPK and MTOR activator 1	1.528	0.00314
LARP6	La ribonucleoprotein domain family member 6	-1.884	0.000371
LCA5	LCA5, lebercilin	2.56	0.00093
LCAT	Lecithin-cholesterol acyltransferase	-2.532	3.31E-08
LDHB	Lactate dehydrogenase B	1.767	0.00103
LDHC	Lactate dehydrogenase C	2.285	0.00283
LDHD	Lactate dehydrogenase D	1.965	0.000112
LDLRAD3	Low density lipoprotein receptor class A domain containing 3	-1.977	0.0106
LENG9	Leukocyte receptor cluster member 9	1.557	0.0202
LEP	Leptin	2.757	0.0000702
LETM1	Leucine zipper and EF-hand containing transmembrane protein 1	1.58	0.0306
LGALS3BP	Galectin 3 binding protein	1.509	0.0258
LHPP	Phospholysine phosphohistidine inorganic pyrophosphate phosphatase	1.928	0.0000379
LIPA	Lipase A, lysosomal acid type	1.72	0.000189
LMO1	LIM domain only 1	1.599	0.0313
LPCAT2	Lysophosphatidylcholine acyltransferase 2	1.783	0.0157
LPCAT3	Lysophosphatidylcholine acyltransferase 3	-1.755	0.00602
LRAT	Lecithin retinol acyltransferase	4.349	0.00126
LRRC17	Leucine rich repeat containing 17	1.908	0.00245
LRRC20	Leucine rich repeat containing 20	1.553	0.0255
LRRC3	Leucine rich repeat containing 3	-1.853	0.0318
LRRC8E	Leucine rich repeat containing 8 VRAC subunit E	2.287	0.00503
LRRN4CL	LRRN4 C-terminal like	1.574	0.00232
LSR	Lipolysis stimulated lipoprotein receptor	-2.366	0.0000375

LSS	Lanosterol synthase	1.563	0.0396
LXN	Latexin	-1.684	0.00709
LY6E	Lymphocyte antigen 6 family member E	5.461	0.00000446
LYNX1	Ly6/neurotoxin 1	3.151	0.00000144
LZTFL1	Leucine zipper transcription factor like 1	-1.504	0.0129
MAATS1	MYCBP associated and testis expressed 1	-1.739	0.0241
MAFF	MAF bZIP transcription factor F	-2.501	0.000166
MAGED2	MAGE family member D2	-1.765	0.000000754
MAGIX	MAGI family member, X-linked	1.829	0.0000171
MALT1	MALT1 paracaspase	-1.512	0.00513
MAMLD1	Mastermind like domain containing 1	2.107	0.0154
MAP1LC3A	Microtubule associated protein 1 light chain 3 alpha	1.622	0.0313
MAP2K6	Mitogen-activated protein kinase kinase 6	1.591	0.0155
MAP7	Microtubule associated protein 7	1.741	0.0277
MAPT	Microtubule associated protein tau	-2.477	0.00116
MARCH2	Membrane associated ring-CH-type finger 2	1.923	0.0000232
MASP1	Mannan binding lectin serine peptidase 1	1.879	0.00151
MAT2A	Methionine adenosyltransferase 2A	-1.619	0.0126
MBLAC1	Metallo-beta-lactamase domain containing 1	1.516	0.0274
MCHR1	Melanin concentrating hormone receptor 1	2.343	0.01
MCUR1	Mitochondrial calcium uniporter regulator 1	1.674	0.000383
MDFI	MyoD family inhibitor	-2.119	0.0116
MDM1	Mdm1 nuclear protein	-1.633	0.00406
ME1	Malic enzyme 1	1.701	0.0327
MEGF10	Multiple EGF like domains 10	2.241	0.0458
MERTK	MER proto-oncogene, tyrosine kinase	-2.453	0.000186
METRNL	Meteorin like, glial cell differentiation regulator	2.043	0.000113
MFAP2	Microfibril associated protein 2	1.557	0.0351
MFSD2B	Major facilitator superfamily domain containing 2B	1.692	0.0101
MGP	Matrix Gla protein	3.217	1.87E-08
MGST1	Microsomal glutathione S-transferase 1	1.952	0.00000224
MGST2	Microsomal glutathione S-transferase 2	-1.504	0.0318
MLF1	Myeloid leukemia factor 1	-2.986	1.99E-14
MMD	Monocyte to macrophage differentiation associated	1.548	0.0134
MMP15	Matrix metallopeptidase 15	1.642	0.00458

MMP25	Matrix metallopeptidase 25	1.648	0.0265
MOB3B	MOB kinase activator 3B	-1.763	0.00662
MOG	Myelin oligodendrocyte glycoprotein	3.102	0.000643
MOK	MOK protein kinase	-1.681	0.0224
MORN1	MORN repeat containing 1	-1.539	0.035
MOSPD1	Motile sperm domain containing 1	1.507	0.0358
MPC2	Mitochondrial pyruvate carrier 2	1.663	0.0348
MPV17L	MPV17 mitochondrial inner membrane protein like	2.244	0.000794
MRAS	Muscle RAS oncogene homolog	1.566	0.00277
MREG	Melanoregulin	1.655	0.0287
MROH6	Maestro heat like repeat family member 6	1.699	0.012
MRPL34	Mitochondrial ribosomal protein L34	1.627	0.0206
MRPL51	Mitochondrial ribosomal protein L51	1.509	0.0255
MSI2	Musashi RNA binding protein 2	1.61	0.0396
MSRA	Methionine sulfoxide reductase A	1.721	0.000175
MT-ATP6	ATP synthase F0 subunit 6	2.002	0.000422
MT-CO1	Cytochrome c oxidase subunit I	2.407	0.0000893
MT-CO2	Cytochrome c oxidase subunit II	2.193	0.00113
MT-CO3	Cytochrome c oxidase III	2.207	0.00023
MT-CYB	Cytochrome b	2.14	0.00139
MT-ND1	NADH dehydrogenase, subunit 1 (complex I)	1.984	0.000177
MT-ND2	MTND2	1.727	0.00409
MT-ND3	NADH dehydrogenase, subunit 3 (complex I)	2.518	0.0000224
MT-ND4	NADH dehydrogenase, subunit 4 (complex I)	2.189	0.000069
MT-ND4L	NADH dehydrogenase, subunit 4L (complex I)	2.364	0.00496
MT-ND5	NADH dehydrogenase, subunit 5 (complex I)	1.972	0.00236
MTA3	Metastasis associated 1 family member 3	-1.522	0.00139
MTERF2	Mitochondrial transcription termination factor 2	2.969	0.0000113
MTFMT	Mitochondrial methionyl-tRNA formyltransferase	1.655	0.00783
MTHFD1	Methylenetetrahydrofolate dehydrogenase, cyclohydrolase and formyltetrahydrofolate synthetase 1	1.813	0.0000089
MTMR12	Myotubularin related protein 12	-1.902	0.0287
MUSTN1	Musculoskeletal, embryonic nuclear protein 1	-3.686	0.00023
MXRA5	Matrix remodeling associated 5	1.933	0.00241
MYADM	Myeloid associated differentiation marker	-1.693	0.000993
MYC	MYC proto-oncogene, bHLH transcription factor	-2.287	0.0000877

MYO1A	Myosin IA	-1.841	0.00663
MYPOP	Myb related transcription factor, partner of profilin	1.596	0.00373
NAB2	NGFI-A binding protein 2	-1.593	0.00116
NADK	NAD kinase	1.514	0.00409
NAP1L5	Nucleosome assembly protein 1 like 5	1.546	0.00693
NCAPH	Non-SMC condensin I complex subunit H	-1.505	0.00685
NCK1	NCK adaptor protein 1	-1.506	0.00497
NCS1	Neuronal calcium sensor 1	-1.626	0.000035
NDUFA1	NADH:ubiquinone oxidoreductase subunit A1	1.916	0.0000231
NDUFA10	NADH:ubiquinone oxidoreductase subunit A10	2.002	0.0018
NDUFA13	NADH:ubiquinone oxidoreductase subunit A13	1.783	0.0121
NDUFA2	NADH:ubiquinone oxidoreductase subunit A2	1.687	0.00867
NDUFA4	NDUFA4, mitochondrial complex associated	1.666	0.0227
NDUFAB1	NADH:ubiquinone oxidoreductase subunit AB1	1.692	0.0399
NDUFAF5	NADH:ubiquinone oxidoreductase complex assembly factor 5	1.973	0.0197
NDUFAF7	NADH:ubiquinone oxidoreductase complex assembly factor 7	1.752	0.000166
NDUFB3	NADH:ubiquinone oxidoreductase subunit B3	1.521	0.0374
NDUFS2	NADH:ubiquinone oxidoreductase core subunit S2	1.719	0.00614
NDUFS3	NADH:ubiquinone oxidoreductase core subunit S3	1.659	0.0354
NDUFS6	NADH:ubiquinone oxidoreductase subunit S6	1.737	0.0249
NDUFV1	NADH:ubiquinone oxidoreductase core subunit V1	2.138	0.00159
Nek10	NIMA (never in mitosis gene a)- related kinase 10	-1.892	0.0314
NEK4	NIMA related kinase 4	-1.587	0.0000474
NFKBIA	NFKB inhibitor alpha	-2.176	0.000174
NIT1	Nitrilase 1	1.578	0.011
NKD2	Naked cuticle homolog 2	1.916	0.00349
NMB	Neuromedin B	-7.157	0.000057
NMNAT2	Nicotinamide nucleotide adenylyltransferase 2	1.885	0.0000813
NOBOX	NOBOX oogenesis homeobox	-2.205	0.000449
NOSTRIN	Nitric oxide synthase trafficking	-1.638	0.012
NPAS3	Neuronal PAS domain protein 3	-2.282	0.00513
NPC2	NPC intracellular cholesterol transporter 2	1.502	0.00845
NPR1	Natriuretic peptide receptor 1	-1.853	0.00017
NQO1	NAD(P)H quinone dehydrogenase 1	2.409	7.66E-08
Nradd	Neurotrophin receptor associated death domain	-1.578	0.0151

NRBP2	Nuclear receptor binding protein 2	-1.721	0.0000926
NRIP3	Nuclear receptor interacting protein 3	-2.789	0.000906
NSDHL	NAD(P) dependent steroid dehydrogenase-like	1.569	0.00101
NT5DC1	5'-nucleotidase domain containing 1	1.52	0.00295
NTN5	Netrin 5	-3.115	0.0000734
OGA	O-GlcNAcase	-1.574	0.000333
OGFOD3	2-oxoglutarate and iron dependent oxygenase domain containing 3	1.89	0.000903
OGT	O-linked N-acetylglucosamine (GlcNAc) transferase	-1.635	0.00249
OPRL1	Opioid related nociceptin receptor 1	-2.838	0.00000109
ORC1	Origin recognition complex subunit 1	1.635	0.0348
ORM1	Orosomucoid 1	9.407	3.73E-08
OSBP2	Oxysterol binding protein 2	2.478	0.0273
OXCT1	3-oxoacid CoA-transferase 1	1.829	0.00047
P2RX4	Purinergic receptor P2X 4	-1.511	0.000525
P2RX6	Purinergic receptor P2X 6	1.968	0.0234
P2RY2	Purinergic receptor P2Y2	-1.875	0.00261
PACS1	Phosphofurin acidic cluster sorting protein 1	-1.704	0.00503
PANK3	Pantothenate kinase 3	1.885	0.0476
PARD6B	Par-6 family cell polarity regulator beta	-2.166	0.0242
PARM1	Prostate androgen-regulated mucin-like protein 1	3.209	0.00000656
PARP3	Poly(ADP-ribose) polymerase family member 3	-1.663	0.0013
PATJ	PATJ, crumbs cell polarity complex component	-1.547	0.0158
PCCB	Propionyl-CoA carboxylase subunit beta	1.598	0.0011
PCK1	Phosphoenolpyruvate carboxykinase 1	2.351	0.00332
PCYT2	Phosphate cytidylyltransferase 2, ethanolamine	1.726	0.0225
PDE5A	Phosphodiesterase 5A	1.787	0.00959
PDF	Peptide deformylase, mitochondrial	1.921	0.0272
PDHA1	Pyruvate dehydrogenase E1 alpha 1 subunit	1.63	0.0214
PDSS1	Decaprenyl diphosphate synthase subunit 1	-1.981	0.00398
PDXP	Pyridoxal phosphatase	1.901	0.000122
PDZRN3	PDZ domain containing ring finger 3	2.071	0.00102
PEBP1	Phosphatidylethanolamine binding protein 1	2.151	0.000000202
PENK	Proenkephalin	4.361	9.25E-08
PER1	Period circadian regulator 1	-1.842	0.00472
PER2	Period circadian regulator 2	-2.257	0.0028

PFKFB2	6-phosphofructo-2-kinase/fructose-2,6-biphosphatase 2	1.903	0.0000168
PGAM1	Phosphoglycerate mutase 1	1.523	0.0123
PGD	Phosphogluconate dehydrogenase	1.717	0.00476
PGF	Placental growth factor	2.404	0.0116
PGLYRP2	Peptidoglycan recognition protein 2	-3.307	0.00000198
PHKA2	Phosphorylase kinase regulatory subunit alpha 2	-1.712	0.000891
PHLDB3	Pleckstrin homology like domain family B member 3	-1.764	0.00312
PIGS	Phosphatidylinositol glycan anchor biosynthesis class S	1.754	0.00000112
PIR	Pirin	1.64	0.000668
PITPNM1	Phosphatidylinositol transfer protein membrane associated 1	-1.592	0.000181
PKD2L1	Polycystin 2 like 1, transient receptor potential cation channel	4.764	1.9E-09
PKNOX1	PBX/knotted 1 homeobox 1	-1.674	0.00014
PLA1A	Phospholipase A1 member A	-1.509	0.0337
PLA2G5	Phospholipase A2 group V	4.817	0.00000015
PLAUR	Plasminogen activator, urokinase receptor	-1.595	0.0103
PLCD1	Phospholipase C delta 1	-1.892	0.00000181
PLCH2	Phospholipase C eta 2	-1.621	0.0223
PLD5	Phospholipase D family member 5	-2.127	0.00584
PLEKHB1	Pleckstrin homology domain containing B1	2.9	0.00452
PLIN5	Perilipin 5	1.817	0.0205
PLOD2	Procollagen-lysine,2-oxoglutarate 5-dioxygenase 2	-2.178	0.000247
PLP2	Proteolipid protein 2	-1.64	0.000369
PLXDC1	Plexin domain containing 1	2.412	2.39E-08
PLXNA4	Plexin A4	-2.334	0.0206
PMEPA1	Prostate transmembrane protein, androgen induced 1	-1.768	0.00105
PNPO	Pyridoxamine 5'-phosphate oxidase	1.846	0.00586
POLA2	DNA polymerase alpha 2, accessory subunit	-1.987	0.00147
POLDIP2	DNA polymerase delta interacting protein 2	1.533	0.00503
POLE	DNA polymerase epsilon, catalytic subunit	2.037	0.0195
Poln	DNA polymerase N	1.93	0.00123
POLR2E	RNA polymerase II subunit E	1.657	0.00286
POLR3G	RNA polymerase III subunit G	1.96	0.0135
POLR3H	RNA polymerase III subunit H	2.01	0.00387
POLR3K	RNA polymerase III subunit K	1.641	0.00626
PPDPF	Pancreatic progenitor cell differentiation and proliferation factor	-1.932	0.0038

PPIP5K1	Diphosphoinositol pentakisphosphate kinase 1	1.619	0.00871
PPP1R2	Protein phosphatase 1 regulatory inhibitor subunit 2	1.545	0.0243
PPP5C	Protein phosphatase 5 catalytic subunit	-2.438	0.000186
PPT1	Palmitoyl-protein thioesterase 1	1.535	0.0000202
PRDM16	PR/SET domain 16	-1.9	0.0383
PRDX1	Peroxiredoxin 1	1.853	0.000788
PRDX3	Peroxiredoxin 3	1.618	0.00209
PRNP	Prion protein	1.532	0.00383
PROX2	Prospero homeobox 2	1.789	0.0143
PROZ	Protein Z, vitamin K dependent plasma glycoprotein	-2.227	0.00249
PRPS2	Phosphoribosyl pyrophosphate synthetase 2	1.764	0.0358
PRR19	Proline rich 19	-1.61	0.0398
PRR5L	Proline rich 5 like	-1.502	0.00237
PRRT4	Proline rich transmembrane protein 4	3.362	7.58E-15
PRSS23	Serine protease 23	-2.019	0.00198
PRUNE2	Prune homolog 2	3.376	0.0078
PSPH	Phosphoserine phosphatase	-2.075	0.00213
PTGR1	Prostaglandin reductase 1	1.603	0.0482
PTGS2	Prostaglandin-endoperoxide synthase 2	-3.065	0.0155
PTPRD	Protein tyrosine phosphatase, receptor type D	-2.201	0.0217
PTPRS	Protein tyrosine phosphatase, receptor type S	-1.67	0.00503
PTX3	Pentraxin 3	-102.543	1.56E-19
PVALB	Parvalbumin	8.43	6.44E-11
PWWP2B	PWWP domain containing 2B	-1.601	0.00698
QPCT	Glutaminyl-peptide cyclotransferase	1.932	0.000000115
RAB26	RAB26, member RAS oncogene family	1.726	0.0264
RAB27B	RAB27B, member RAS oncogene family	-2.037	0.0398
RAB3A	RAB3A, member RAS oncogene family	1.96	0.00152
RAD51B	RAD51 paralog B	2.303	0.000142
RALGAPA2	Ral GTPase activating protein catalytic alpha subunit 2	-1.763	0.00435
RARRES2	Retinoic acid receptor responder 2	-2.54	0.00192
RASA3	RAS p21 protein activator 3	-2.235	2.85E-09
RASAL1	RAS protein activator like 1	-1.607	0.00516
RASD1	Ras related dexamethasone induced 1	-1.508	0.0316
RASEF	RAS and EF-hand domain containing	2.222	0.000906

RASGRP2	RAS guanyl releasing protein 2	-1.737	0.00203
RASL11B	RAS like family 11 member B	-2.261	0.0000278
RASSF7	Ras association domain family member 7	-1.705	0.0131
RBM47	RNA binding motif protein 47	3.13	0.00000756
RBMX2	RNA binding motif protein X-linked 2	-2.347	0.00000506
RCN3	Reticulocalbin 3	1.605	0.0179
RDH10	Retinol dehydrogenase 10	-1.62	0.0132
RDH16	Retinol dehydrogenase 16	-4.707	0.00189
RET	Ret proto-oncogene	1.741	0.0358
RETSAT	Retinol saturase	2.172	0.00151
REV3L	REV3 like, DNA directed polymerase zeta catalytic subunit	-1.712	0.00374
REX1BD	Required for excision 1-B domain containing	1.579	0.0244
REXO2	RNA exonuclease 2	1.68	0.00832
RGD1561161	Similar to BC067074 protein	-1.627	0.0214
RGN	Regucalcin	1.589	0.0000503
RGS1	Regulator of G protein signaling 1	-2.883	0.0135
RGS3	Regulator of G protein signaling 3	-1.679	0.0011
RHOB	Ras homolog family member B	-1.681	0.00958
RIBC1	RIB43A domain with coiled-coils 1	-1.832	0.0241
RIDA	Reactive intermediate imine deaminase A homolog	1.519	0.0112
RIMKLA	Ribosomal modification protein rimK like family member A	3.998	0.000000031
RIMKLB	Ribosomal modification protein rimK like family member B	-1.549	0.0276
RNASE1	Ribonuclease A family member 1, pancreatic	1.7	0.0332
RNASEH2A	Ribonuclease H2 subunit A	1.66	0.00172
RNF125	Ring finger protein 125	2.116	0.000486
RNF128	Ring finger protein 128, E3 ubiquitin protein ligase	3.011	0.000000126
RNF157	Ring finger protein 157	1.654	0.0107
RNF217	Ring finger protein 217	-2.455	0.0000381
ROBO1	Roundabout guidance receptor 1	1.732	0.00937
ROBO2	Roundabout guidance receptor 2	-2.554	0.00000751
RORC	RAR related orphan receptor C	-2.21	0.00222
RPL3	Ribosomal protein L3	-1.536	0.0036
RPLP0	Ribosomal protein lateral stalk subunit P0	-1.604	0.0091
RTL1	Retrotransposon Gag like 1	-5.673	0.000000884
RYR1	Ryanodine receptor 1	-1.529	0.00394

S100A10	S100 calcium binding protein A10	2.054	0.0000288
S100B	S100 calcium binding protein B	2.398	6.03E-11
S1PR3	Sphingosine-1-phosphate receptor 3	-2.401	0.000915
SAMD11	Sterile alpha motif domain containing 11	-1.744	0.0089
SAMSN1	SAM domain, SH3 domain and nuclear localization signals 1	2.342	0.0000085
SCD	Stearoyl-CoA desaturase	3.395	0.0216
SCN9A	Sodium voltage-gated channel alpha subunit 9	-2.695	0.00011
SCPEP1	Serine carboxypeptidase 1	1.597	0.000422
SCUBE2	Signal peptide, CUB domain and EGF like domain containing 2	-2.028	0.000248
SCUBE3	Signal peptide, CUB domain and EGF like domain containing 3	3.267	0.00693
SDC4	Syndecan 4	-1.784	0.0000172
SDCBP2	Syndecan binding protein 2	-2.02	0.00199
SDHA	Succinate dehydrogenase complex flavoprotein subunit A	2.162	0.000183
SDHC	Succinate dehydrogenase complex subunit C	1.507	0.0155
SDS	Serine dehydratase	1.638	0.0398
Sectm1b	Secreted and transmembrane 1B	-3.722	0.000256
SEL1L3	SEL1L family member 3	10.15	9.12E-15
SELENOW	Selenoprotein W	1.86	0.00218
SEPT6	Septin 6	-2.027	0.00000175
SERF2	Small EDRK-rich factor 2	1.786	0.00167
SERINC2	Serine incorporator 2	1.848	0.0478
SERPINA5	Serpin family A member 5	11.098	6.99E-24
SERPINB1	Serpin family B member 1	-1.53	0.0051
SERPINF1	Serpin family F member 1	1.701	0.0000373
SESN1	Sestrin 1	-1.55	0.0175
SETD9	SET domain containing 9	-1.58	0.00112
SFT2D3	SFT2 domain containing 3	-1.6	0.0135
SGCA	Sarcoglycan alpha	1.904	0.0148
SGK1	Serum/glucocorticoid regulated kinase 1	-1.701	0.00783
SGTB	Small glutamine rich tetratricopeptide repeat containing beta	-2.044	0.0104
SH2B2	SH2B adaptor protein 2	-1.738	0.0366
SH2D7	SH2 domain containing 7	2.812	0.00000022
SH3BGRL2	SH3 domain binding glutamate rich protein like 2	-1.871	0.0478
SH3GL2	SH3 domain containing GRB2 like 2, endophilin A1	1.552	0.0337
SHISAL2A	Shisa like 2A	-2.431	0.00234

SHROOM1	Shroom family member 1	-1.574	0.0143
SINHCAF	SIN3-HDAC complex associated factor	-1.536	0.00407
SIRPA	Signal regulatory protein alpha	1.889	2.39E-08
SIRT3	Sirtuin 3	1.793	0.000281
SKAP1	Src kinase associated phosphoprotein 1	-2.306	0.0000185
SLC12A4	Solute carrier family 12 member 4	-1.675	0.000267
SLC16A1	Solute carrier family 16 member 1	1.86	0.0224
SLC16A11	Solute carrier family 16 member 11	-2.36	0.000000102
SLC16A14	Solute carrier family 16 member 14	-2.241	0.00368
SLC19A3	Solute carrier family 19 member 3	2.36	0.00000598
SLC1A1	Solute carrier family 1 member 1	4.13	0.00000102
SLC1A5	Solute carrier family 1 member 5	-1.758	0.033
SLC22A23	Solute carrier family 22 member 23	1.718	0.0447
SLC22A3	Solute carrier family 22 member 3	1.955	0.00462
SLC23A3	Solute carrier family 23 member 3	-2.094	0.000919
Slc25a1	Solute carrier family 25 (mitochondrial carrier, citrate transporter), member 1	2.973	0.0000652
SLC25A20	Solute carrier family 25 member 20	1.644	0.00952
SLC25A25	Solute carrier family 25 member 25	-1.553	0.0388
SLC25A4	Solute carrier family 25 member 4	2.19	0.0102
SLC26A8	Solute carrier family 26 member 8	3.229	0.000902
SLC27A3	Solute carrier family 27 member 3	-4.629	2.96E-09
SLC28A1	Solute carrier family 28 member 1	2.357	0.00314
SLC29A2	Solute carrier family 29 member 2	-1.664	0.0273
SLC35G2	Solute carrier family 35 member G2	-1.7	0.00474
SLC38A3	Solute carrier family 38 member 3	-2.586	0.00414
SLC39A13	Solute carrier family 39 member 13	-1.572	0.000357
SLC44A3	Solute carrier family 44 member 3	2.187	0.000384
SLC45A4	Solute carrier family 45 member 4	2.174	0.0000656
SLC46A3	Solute carrier family 46 member 3	1.774	0.00000294
SLC4A10	Solute carrier family 4 member 10	2.423	0.00593
SLC4A11	Solute carrier family 4 member 11	-2.415	0.000487
SLC4A4	Solute carrier family 4 member 4	4.255	0.0000168
SLC4A5	Solute carrier family 4 member 5	2.334	0.00793
SLC50A1	Solute carrier family 50 member 1	-1.644	0.000142
SLC5A6	Solute carrier family 5 member 6	1.979	0.00519

SLC6A9	Solute carrier family 6 member 9	-1.738	0.00129
SMARCA1	SWI/SNF related, matrix associated, actin dependent regulator of chromatin, subfamily a, member 1	-1.746	0.00148
SMTNL2	Smoothelin like 2	1.537	0.0298
SNAPC1	Small nuclear RNA activating complex polypeptide 1	-2.606	0.000000703
SNAPC5	Small nuclear RNA activating complex polypeptide 5	1.936	0.00000224
SNCG	Synuclein gamma	-2.272	0.0137
SNN	Stannin	1.567	0.00578
SOBP	Sine oculis binding protein homolog	2.439	0.00000207
SOCS1	Suppressor of cytokine signaling 1	-1.921	0.00319
SOCS2	Suppressor of cytokine signaling 2	-1.979	0.00000188
SOD1	Superoxide dismutase 1	1.585	0.00044
SOD2	Superoxide dismutase 2	1.853	0.000523
SORCS2	Sortilin related VPS10 domain containing receptor 2	-1.779	0.0483
SORD	Sorbitol dehydrogenase	1.921	0.00000311
SORT1	Sortilin 1	1.717	0.00000746
SOX17	SRY-box 17	-1.521	0.0332
SPARC	Secreted protein acidic and cysteine rich	1.644	0.000357
SPATA6	Spermatogenesis associated 6	-1.617	0.0145
SPEF1	Sperm flagellar 1	2.774	0.0000652
SPEF2	Sperm flagellar 2	-6.933	4.83E-15
SPINT2	Serine peptidase inhibitor, Kunitz type 2	-1.664	0.000777
SPON2	Spondin 2	1.583	0.03
SPTLC3	Serine palmitoyltransferase long chain base subunit 3	1.576	0.0395
SRC	SRC proto-oncogene, non-receptor tyrosine kinase	-1.551	0.0136
SRCIN1	SRC kinase signaling inhibitor 1	-1.971	0.00553
SRD5A1	Steroid 5 alpha-reductase 1	2.097	0.00000031
SSC5D	Scavenger receptor cysteine rich family member with 5 domains	1.503	0.000893
SSR3	Signal sequence receptor subunit 3	-1.518	0.00051
SSUH2	Ssu-2 homolog (C. elegans)	-2.571	0.0011
STAT6	Signal transducer and activator of transcription 6	-2.159	5.13E-09
STK10	Serine/threonine kinase 10	-1.502	0.0244
STK40	Serine/threonine kinase 40	1.596	0.00919
STS	Steroid sulfatase	4.584	0.00000144
STX12	Syntaxin 12	-1.964	0.000175
STX17	Syntaxin 17	-1.783	0.000000897

STXBP2	Syntaxin binding protein 2	-1.862	0.000000378
SULF1	Sulfatase 1	1.788	0.0109
Sult1a1	Sulfotransferase family 1A, phenol-preferring, member 1	1.565	0.0165
SULT1C4	Sulfotransferase family 1C member 4	-2.612	0.00996
SWT1	SWT1, RNA endoribonuclease homolog	-1.773	0.0000203
SYN2	Synapsin II	1.565	0.0000494
SYNPO2	Synaptopodin 2	-1.595	0.04
SYT17	Synaptotagmin 17	-2.898	0.00558
TAF1D	TATA-box binding protein associated factor, RNA polymerase I subunit D	-1.773	0.0000321
TALDO1	Transaldolase 1	1.909	0.00157
TARSL2	Threonyl-tRNA synthetase like 2	-2.348	0.00157
TBC1D20	TBC1 domain family member 20	1.765	0.00000606
TBKBP1	TBK1 binding protein 1	-1.526	0.00373
TBX19	T-box 19	-1.589	0.0214
TCAF1	TRPM8 channel associated factor 1	-1.511	0.000372
TCAF2	TRPM8 channel associated factor 2	-1.682	0.00729
TCF21	Transcription factor 21	-2.887	0.000878
TCF23	Transcription factor 23	2.127	0.0000293
TCIM	Transcriptional and immune response regulator	-1.807	0.00324
Tdh	L-threonine dehydrogenase	7.054	0.000239
TDRD5	Tudor domain containing 5	2.625	0.00574
TECR	Trans-2,3-enoyl-CoA reductase	1.53	0.0322
TEKT3	Tektin 3	-1.935	0.0478
TENM3	Teneurin transmembrane protein 3	1.697	0.0175
TF	Transferrin	2.921	0.000777
TGM3	Transglutaminase 3	7.144	0.0000346
TGM5	Transglutaminase 5	3.074	0.00196
Tha1	Threonine aldolase 1	1.559	0.00472
THBS2	Thrombospondin 2	2.053	0.00496
THEM6	Thioesterase superfamily member 6	2.084	0.00458
THRB	Thyroid hormone receptor beta	2.005	0.000308
THRSP	Thyroid hormone responsive	3.748	3.91E-12
THY1	Thy-1 cell surface antigen	1.728	0.000992
TIGAR	TP53 induced glycolysis regulatory phosphatase	1.606	0.0276
TIMM23	Translocase of inner mitochondrial membrane 23	1.72	0.00399

TIMP2	TIMP metallopeptidase inhibitor 2	-1.536	0.0339
TKT	transketolase	1.543	0.0313
TLN2	Talin 2	1.588	0.0329
TLNRD1	Talin rod domain containing 1	-1.666	0.000877
TLR8	Toll like receptor 8	2.459	0.000405
TMBIM6	Transmembrane BAX inhibitor motif containing 6	1.909	1.44E-09
TMC6	Transmembrane channel like 6	-1.546	0.000118
TMC8	Transmembrane channel like 8	-1.794	0.000902
TMEFF2	Transmembrane protein with EGF like and two follistatin like domains 2	2.054	0.00416
TMEM106C	Transmembrane protein 106C	1.777	0.0126
TMEM107	Transmembrane protein 107	-1.697	0.0453
TMEM110-MUSTN1	TMEM110-MUSTN1 readthrough	2.173	0.00000452
TMEM119	Transmembrane protein 119	2.43	0.000929
TMEM132C	Transmembrane protein 132C	-2.636	0.0000185
TMEM132E	Transmembrane protein 132E	3.231	0.00000224
TMEM160	Transmembrane protein 160	2.04	0.00249
TMEM163	Transmembrane protein 163	-1.991	0.00155
TMEM187	Transmembrane protein 187	1.505	0.00584
TMEM202	Transmembrane protein 202	3.302	0.0000162
TMEM236	Transmembrane protein 236	-4.111	0.0167
TMEM249	Transmembrane protein 249	1.61	0.012
TMEM254	Transmembrane protein 254	-2.3	2.11E-08
TNFAIP6	TNF alpha induced protein 6	-2.949	0.00000224
TNFRSF12A	TNF receptor superfamily member 12A	-1.894	0.0167
TNFRSF4	TNF receptor superfamily member 4	-1.728	0.0227
TNFRSF6B	TNF receptor superfamily member 6b	-2.957	0.00156
TNNI2	Troponin I2, fast skeletal type	-7.543	2.73E-14
TNS2	Tensin 2	-1.648	0.0000901
TOM1L1	Target of myb1 like 1 membrane trafficking protein	-1.804	0.000102
Tomm5	Translocase of outer mitochondrial membrane 5	2.002	0.00326
TP53INP2	Tumor protein p53 inducible nuclear protein 2	2.032	0.0114
TRAF3IP2	TRAF3 interacting protein 2	-1.726	0.0000723
TRAF4	TNF receptor associated factor 4	1.661	0.00385
TRAP1	TNF receptor associated protein 1	1.652	0.00887

TRIP13	Thyroid hormone receptor interactor 13	-1.517	0.0195
TSPAN4	Tetraspanin 4	-1.712	0.000648
TSPAN7	Ttraspanin 7	2.317	9.48E-11
Tstd3	Thiosulfate sulfurtransferase (rhodanese)-like domain containing 3	1.725	0.0301
TTC12	Tetratricopeptide repeat domain 12	-2.579	0.000193
TTC19	Tetratricopeptide repeat domain 19	1.557	0.00752
TTC25	Tetratricopeptide repeat domain 25	5.97	0.00000012
TTC9	Tetratricopeptide repeat domain 9	-1.71	0.0175
TTN	Titin	-2.92	0.01
TTPA	Alpha tocopherol transfer protein	-1.974	0.0337
TUBA1B	Tubulin alpha 1b	-1.664	0.0242
TUBB	Tubulin beta class I	-2.067	0.0000657
TUBB4B	Tubulin beta 4B class IVb	-1.569	0.0236
TUBB6	Tubulin beta 6 class V	-1.535	0.0091
TWIST2	Twist family bHLH transcription factor 2	1.745	0.0000507
TXN2	Thioredoxin 2	1.675	0.0138
TXNIP	Thioredoxin interacting protein	-1.703	0.0175
TXNRD2	Thioredoxin reductase 2	1.552	0.0041
TYRP1	Tyrosinase related protein 1	3.69	0.00011
UBC	Ubiquitin C	1.89	0.000000194
UBXN11	UBX domain protein 11	-1.84	0.00677
UCHL1	Tbiquitin C-terminal hydrolase L1	-2.95	0.00515
UCHL3	Ubiquitin C-terminal hydrolase L3	1.677	0.00954
UQCR10	Ubiquinol-cytochrome c reductase, complex III subunit X	2.022	0.00133
UQCR11	Ubiquinol-cytochrome c reductase, complex III subunit XI	1.897	0.00519
UQCRC1	Ubiquinol-cytochrome c reductase core protein 1	1.776	0.00941
UQCRFS1	Ubiquinol-cytochrome c reductase, Rieske iron-sulfur polypeptide 1	1.659	0.022
VAMP8	Vesicle associated membrane protein 8	1.634	0.00551
VAV3	Vav guanine nucleotide exchange factor 3	1.728	0.0195
VCAM1	Vascular cell adhesion molecule 1	1.755	0.00301
VCAN	Versican	-1.959	0.0119
VEGFA	Vascular endothelial growth factor A	-1.593	0.0191
VIM	Vimentin	-1.509	0.0292
VIPR2	Vasoactive intestinal peptide receptor 2	1.742	0.0238
VLDLR	Very low density lipoprotein receptor	1.606	0.012

VSTM2L	V-set and transmembrane domain containing 2 like	-1.702	0.0132
VWA2	Von Willebrand factor A domain containing 2	2.037	0.0038
WARS2	Tryptophanyl tRNA synthetase 2, mitochondrial	-1.657	0.00000756
WFS1	Wolframin ER transmembrane glycoprotein	-1.62	0.00701
WISP2	WNT1 inducible signaling pathway protein 2	2.038	0.00698
WNK4	WNK lysine deficient protein kinase 4	-1.691	0.00126
WNT11	Wnt family member 11	1.603	0.00832
XDH	Xanthine dehydrogenase	-1.7	0.00775
XK	X-linked Kx blood group	2.029	0.00066
XPO1	Exportin 1	-1.963	8.06E-08
YBX2	Y-box binding protein 2	-1.595	0.00468
ZBTB42	Zinc finger and BTB domain containing 42	-1.644	0.00484
Zcchc13	Zinc finger, CCHC domain containing 13	3.435	0.00000411
ZCCHC7	Zinc finger CCHC-type containing 7	-1.521	0.000731
ZDHHC14	Zinc finger DHHC-type containing 14	1.679	0.000152
ZMYND15	Zinc finger MYND-type containing 15	1.562	0.01
ZNF106	Zinc finger protein 106	1.879	0.0051
ZNF217	Zinc finger protein 217	-1.596	0.00962
ZNF277	Zinc finger protein 277	-1.662	0.0137
ZNF280C	Zinc finger protein 280C	-1.865	0.00182
ZNF365	Zinc finger protein 365	1.954	0.02
ZNF385B	Zinc finger protein 385B	-3.665	0.00277
ZNF713	Zinc finger protein 713	-1.644	0.022

[1] Genes highlighted in bold were also differentially expressed in subcutaneous adipose of bull calves fed enhanced diet during early life in English et al. (2018). All genes highlighted followed the same direction of change the current study and that of English et al. (2018), with the exception of *IFIT3*, *KIAA0408*, *PRR5L*, *PSPH*, *SLC29A2*, *SMTNL2*, *TSPAN7* and *WISP2* which followed different direction of effect between the two studies.

[2]fold changes are up or down in High dietary group (HI) compared to Moderate dietary group (MOD)

Supplementary Table 2: Biochemical pathways significantly enriched in visceral adipose of heifer calves offered either a High plane of nutrition or a Moderate plane of nutrition for the first 21 weeks of life.

Pathway name	adj. p-value	Genes
Oxidative Phosphorylation	5.01E-21	*NDUFA10, MT-CYB, COX7B, MT-ND5, COX7A1, UQCR10, MT-ND3, NDUFA1, MT-CO3, MT-ND2, MT-ND1, NDUFA13, UQCR11, MT-ND4L, UQCRFS1, SDHC, MT-ND4, MT-ATP6, UQCRC1, MT-CO1, NDUFB3, SDHA, COX4I2, NDUFV1, NDUFA2, NDUFAB1, Cox6c, NDUFA4, NDUFS2, CYB5A, NDUFS3, NDUFS6, ATP5MF, MT-CO2*
Mitochondrial Dysfunction	2E-19	*NDUFA10, MT-CYB, COX7B, MT-ND5, COX7A1, UQCR10, MT-ND3, NDUFA1, MT-CO3, MT-ND2, PRDX3, MT-ND1, NDUFA13, TXN2, UQCR11, MT-ND4L, UQCRFS1, TXNRD2, SDHC, MT-ND4, MT-ATP6, UQCRC1, MT-CO1, NDUFB3, SDHA, COX4I2, NDUFV1, NDUFA2, PDHA1, NDUFAB1, Cox6c, SOD2, NDUFA4, NDUFS2, CYB5A, NDUFS3, NDUFS6, ATP5MF, MT-CO2, XDH*
Sirtuin Signaling Pathway	5.01E-15	*NDUFA10, MT-CYB, MT-ND5, AKT1, PGAM1, ACSS2, GOT2, MT-ND3, NDUFA1, NQO1, MT-ND2, MT-ND1, NDUFA13, TIMM23, ARNTL, MT-ND4L, UQCRFS1, PCK1, SDHC, MT-ND4, MT-ATP6, G6PD, NDUFB3, SOD1, SDHA, MAP1LC3A, NDUFV1, NDUFA2, PDHA1, NDUFAB1, HIST1H4J, SOD2, NDUFA4, NDUFS2, MYC, NDUFS3, LDHC, SIRT3, SLC25A4, HIF1A, ACLY, TUBA1B, Tomm5, LDHD, NDUFS6, LDHB*
Gluconeogenesis I	6.61E-05	*PGAM1, ENO4, ALDOC, ME1, FBP1, ALDOA, GPI*
STAT3 Pathway	0.000112	*FGFR3, SRC, IL1B, VEGFA, MRAS, IL20RA, CDC25A, IL2RA, IL6R, INSR, SOCS2, CISH, IGF1, NDUFA13, MYC, SOCS1*
Superoxide Radicals Degradation	0.000178	*SOD2, SOD1, TYRP1, NQO1*
Glutathione-mediated Detoxification	0.000275	*GSTM4, GSTA4, MGST2, GSTA1, Gstt3, Gstt1, MGST1*
Glycolysis I	0.000562	*PGAM1, ENO4, ALDOC, FBP1, ALDOA, GPI*
Valine Degradation I	0.000646	*SDS, HIBADH, ACADSB, ABAT, ECHS1*
NRF2-mediated Oxidative Stress Response	0.000759	*FGFR3, DNAJC6, IRS2, AKT1, MRAS, SOD1, MGST2, NQO1, PRDX1, MGST1, DNAJB5, MAFF, AKR7A2, SOD2, CDC34, GSTM4, GSTA4, GSTA1, MAP2K6*
Pentose Phosphate Pathway	0.000759	*TKT, PGD, G6PD, TALDO1*
Aryl Hydrocarbon Receptor Signaling	0.000813	*SRC, IL1B, CCND2, ALDH18A1, MGST2, NQO1, MGST1, ALDH4A1, MYC, ALDH2, GSTM4, GSTA4, GSTA1, CTSD, ESR1*
Acute Phase Response Signaling	0.001047	*ITIH4, TF, IL1B, AKT1, SERPINF1, MRAS, ITIH3, IL6R, SOCS2, CRABP2, NFKBIA, SOD2, C2, ORM1, HMOX2, SOCS1, MAP2K6*
Glycine Degradation (Creatine Biosynthesis)	0.001698	*GAMT, GATM*
Fatty Acid β-oxidation I	0.001778	*SDS, ACSBG1, HADH, SLC27A3, IVD, ECHS1*
Isoleucine Degradation I	0.002089	*SDS, ACADSB, ACAT2, ECHS1*
Phenylalanine Degradation IV (Mammalian, via Side	0.002089	*GOT2, ALDH2, HPD, SLC27A3*

Chain)		
Estrogen-Dependent Breast Cancer Signaling	0.003162	*FGFR3, HSD17B14, SRC, IRS2, AKT1, MRAS, HSD17B12, HSD17B11, ESR1, IGF1*
CDP-diacylglycerol Biosynthesis I	0.003162	*AGPAT2, CDS1, LPCAT3, GPAM, LPCAT2*
LXR/RXR Activation	0.004365	*ITIH4, TF, IL1B, ORM1, SCD, CLU, SERPINF1, PTGS2, FASN, HADH, LCAT, ECHS1*
Phosphatidylglycerol Biosynthesis II (Non-plastidic)	0.004571	*AGPAT2, CDS1, LPCAT3, GPAM, LPCAT2*
Sucrose Degradation V (Mammalian)	0.004898	*KHK, ALDOC, ALDOA*
4-hydroxyproline Degradation I	0.005012	*GOT2, ALDH4A1*
14-3-3-mediated Signaling	0.005129	*FGFR3, PLCD1, SRC, IRS2, AKT1, TUBB, MRAS, TUBB6, TUBB4B, VIM, TUBA1B, PLCH2, MAPT*
Superpathway of Cholesterol Biosynthesis	0.00537	*LSS, NSDHL, DHCR24, IDI1, ACAT2*
Ketolysis	0.006761	*ACAT2, BDH1, OXCT1*
Intrinsic Prothrombin Activation Pathway	0.007244	*COL5A3, COL1A2, COL1A1, F13A1, KLK7, COL11A2*
Dendritic Cell Maturation	0.007586	*FGFR3, PLCD1, IRS2, IL1B, AKT1, CD1A, COL5A3, COL1A2, COL1A1, NFKBIA, IL23A, IRF8, HLA-DMB, PLCH2, LEP, COL11A2*
The Visual Cycle	0.008318	*DHRS3, AKR1C3, LRAT, RDH10*
TR/RXR Activation	0.008318	*FGFR3, AKR1C3, THRSP, IRS2, AKT1, HIF1A, FASN, ME1, PCK1, THRB*
Stearate Biosynthesis I (Animals)	0.00912	*ELOVL1, DHCR24, PPT1, ACSBG1, FASN, SLC27A3*
Pentose Phosphate Pathway (Oxidative Branch)	0.009772	*PGD, G6PD*
Phenylethylamine Degradation I	0.009772	*ALDH2, AOC2*
Adipogenesis pathway	0.009772	*AGPAT2, FGFR3, ARNTL, FGF1, TXNIP, AKT1, HIF1A, PER2, PPIP5K1, KLF5, LEP, GTF2H5*
GP6 Signaling Pathway	0.010233	*FGFR3, LAMC2, IRS2, AKT1, COL20A1, LAMB1, COL5A3, COL1A2, COL22A1, COL1A1, COL11A2, RASGRP2*
Hepatic Fibrosis / Hepatic Stellate Cell Activation	0.010715	*FGF1, IL1B, VEGFA, PGF, IL6R, COL5A3, COL1A2, COL22A1, COL1A1, IGF1, VCAM1, TIMP2, COL20A1, LEP, COL11A2*
IL-6 Signaling	0.010965	*FGFR3, IRS2, IL1B, AKT1, VEGFA, TNFAIP6, MRAS, IL6R, SOCS1, COL1A1, MAP2K6, NFKBIA*
Role of JAK2 in Hormone-like Cytokine Signaling	0.012303	*IRS2, SOCS1, SOCS2, SIRPA, SH2B2*
Ethanol Degradation II	0.012303	*ACSS2, ALDH2, ADH6, ADHFE1, ALDH4A1*
Triacylglycerol Biosynthesis	0.012303	*AGPAT2, ELOVL1, LPCAT3, GPAM, LPCAT2, DGAT2*
Cholesterol Biosynthesis I	0.014791	*LSS, NSDHL, DHCR24*
Cholesterol Biosynthesis II (via 24,25-dihydrolanosterol)	0.014791	*LSS, NSDHL, DHCR24*
Cholesterol Biosynthesis III (via Desmosterol)	0.014791	*LSS, NSDHL, DHCR24*
FXR/RXR Activation	0.015488	*ITIH4, TF, IL1B, AKT1, ORM1, CLU, SERPINF1, VLDLR, FASN, LCAT, FBP1*
HIF1α Signaling	0.015488	*MMP25, FGFR3, IRS2, AKT1, LDHC, VEGFA, MRAS, HIF1A, PGF, MMP15, LDHB*
Bladder Cancer Signaling	0.015849	*MMP25, FGFR3, MYC, FGF1, VEGFA, MRAS, PGF, ERBB2, MMP15*

Serine Biosynthesis	0.015849	*PSPH, DUSP26*
Citrulline-Nitric Oxide Cycle	0.015849	*ASS1, ASL*
Folate Polyglutamylation	0.015849	*MTHFD1, FPGS*
Glutathione Redox Reactions I	0.015849	*MGST2, GSTA1, Gstt1, MGST1*
TCA Cycle II (Eukaryotic)	0.015849	*IDH3B, FH, SDHA, SDHC*
Axonal Guidance Signaling	0.015849	*MMP25, AKT1, MRAS, TUBB6, PGF, ADAMTS16, ADAMTS2, RGS3, WNT11, ENPEP, KIF7, ADAMTS17, NTN5, ADAM11, ERBB2, MMP15, NCK1, KALRN, ADAM9, FGFR3, ROBO2, PLCD1, IRS2, VEGFA, TUBB, ROBO1, ADAM19, IGF1, TUBB4B, TUBA1B, PLCH2*
Atherosclerosis Signaling	0.017378	*IL1B, ORM1, CLU, COL5A3, COL1A2, LCAT, COL1A1, TNFRSF12A, COL11A2, PLA2G5, VCAM1*
Androgen Biosynthesis	0.018197	*HSD17B14, SRD5A1, AKR1C3*
Ethanol Degradation IV	0.018197	*ACSS2, ALDH2, TYRP1, ALDH4A1*
AMPK Signaling	0.019953	*FGFR3, SRC, IRS2, AKT1, PFKFB2, MRAS, FASN, INSR, CHRNA3, GYS2, EEF2K, AK2, RAB3A, CPT2, LEP, AK5*
Apelin Liver Signaling Pathway	0.020893	*COL5A3, COL1A2, COL1A1, COL11A2*
Integrin Signaling	0.021878	*FGFR3, SRC, IRS2, AKT1, MRAS, ITGAE, TSPAN4, TSPAN7, TLN2, ITGA7, GRB7, CAPN3, GSN, RHOB, TTN, NCK1*
Superpathway of Citrulline Metabolism	0.021878	*ASS1, ALDH18A1, ASL*
Arginine Biosynthesis IV	0.022909	*ASS1, ASL*
Pyruvate Fermentation to Lactate	0.022909	*LDHC, LDHB*
Urea Cycle	0.022909	*ASS1, ASL*
Glycogen Biosynthesis II (from UDP-D-Glucose)	0.022909	*GYG2,GYS2*
Rapoport-Luebering Glycolytic Shunt	0.022909	*PGAM1, TIGAR*
GDP-mannose Biosynthesis	0.022909	*GMPPB, GPI*
LPS/IL-1 Mediated Inhibition of RXR Function	0.023442	*IL1B, ALDH18A1, MGST2, MGST1, ALDH4A1, XPO1, ACOX1, SULT1C4, ACSBG1, ACOX2, ALDH2, GSTM4, CPT2, GSTA4, SLC27A3, GSTA1*
Angiopoietin Signaling	0.025704	*FGFR3, ANGPT2, IRS2, AKT1, MRAS, NFKBIA, NCK1, GRB7*
Glutaryl-CoA Degradation	0.026303	*ACAT2, HADH, HACD2*
Gap Junction Signaling	0.026303	*FGFR3, PLCD1, SRC, IRS2, AKT1, NPR1, TUBB, GJC2, MRAS, TUBB6, GRIK2, GUCY2D, TUBB4B, TUBA1B, PLCH2*
Type II Diabetes Mellitus Signaling	0.027542	*FGFR3, IRS2, AKT1, ACSBG1, KCNJ11, CACNA1G, INSR, ABCC8, SOCS1, SOCS2, SLC27A3, NFKBIA*
Retinol Biosynthesis	0.02884	*DHRS3, DDHD2, AKR1C3, LRAT, RDH10*
T Helper Cell Differentiation	0.030903	*BCL6, FOXP3, HLA-DMB, IL2RA, STAT6, IL6R, RORC*
γ-linolenate Biosynthesis II (Animals)	0.030903	*CYB5A, ACSBG1, SLC27A3*

Mitochondrial L-carnitine Shuttle Pathway	0.030903	*ACSBG1, CPT2, SLC27A3*
Superpathway of Serine and Glycine Biosynthesis I	0.030903	*PSPH, DUSP26*
Ceramide Biosynthesis	0.030903	*SPTLC3, KDSR*
Pentose Phosphate Pathway (Non-oxidative Branch)	0.030903	*TKT, TALDO1*
Endocannabinoid Cancer Inhibition Pathway	0.031623	*FGFR3, VIM, MYC, SRC, IRS2, AKT1, CCND2, VEGFA, HIF1A, PGF, TWIST2, MAP2K6*
Renal Cell Carcinoma Signaling	0.032359	*FGFR3, IRS2, AKT1, FH, VEGFA, MRAS, HIF1A, UBC*
Pyrimidine Ribonucleotides Interconversion	0.034674	*HNRNPA1, SMARCA1, AK5, PCK1, ENTPD2*
JAK/Stat Signaling	0.034674	*FGFR3, IRS2, AKT1, MRAS, STAT6, SOCS1, SOCS2, CISH*
Superpathway of Inositol Phosphate Compounds	0.038019	*DUSP10, FGFR3, PLCD1, SRC, IRS2, CDC25A, EPHX2, PDXP, INPP5K, ERBB2, PLCH2, HACD2, PPIP5K1, ITPKC, SIRPA, PPP5C*
Pyrimidine Ribonucleotides De Novo Biosynthesis	0.040738	*HNRNPA1, SMARCA1, AK5, PCK1, ENTPD2*
VEGF Signaling	0.040738	*FGFR3, SRC, IRS2, AKT1, VEGFA, MRAS, HIF1A, PGF, EIF2B2*
Acetyl-CoA Biosynthesis III (from Citrate)	0.041687	*ACLY*
Sorbitol Degradation I	0.041687	*SORD*
Methylglyoxal Degradation VI	0.041687	*LDHD*
Histamine Biosynthesis	0.041687	*HDC*
Lanosterol Biosynthesis	0.041687	*LSS*
Insulin Receptor Signaling	0.041687	*FGFR3, IRS2, AKT1, SGK1, INPP5K, MRAS, ACLY, INSR, EIF2B2, NCK1, GYS2*
PEDF Signaling	0.042658	*FGFR3,SOD2,IRS2,AKT1,SERPINF1,MRAS,NFKBIA,ARHGAP22*
IL-4 Signaling	0.045709	*FGFR3,IRS2,AKT1,INPP5K,MRAS,HLA-DMB,STAT6,SOCS1*
Circadian Rhythm Signaling	0.045709	*ARNTL,VIPR2,PER1,PER2*

Supplementary Table 3: Biochemical networks generated within Ingenuity Pathway Analysis (IPA) from genes differentially expressed in visceral adipose of heifer calves offered either a High plane of nutrition or a Moderate plane of nutrition for the first 21 weeks of life.

Network number and function	Genes	Score	Focus Molecules
1.Developmental Disorder, Hereditary Disorder, Metabolic Disease	*ASL, CCDC69, ECHDC2, EPDR1, ETFA, FBXO17, FUT1, GLT8D2, GRIK2, ISCU, IVD, MAATS1, MAPT, MT-ND1, MT-ND4, MT-ND5, NDUFA1, NDUFA10, NDUFA2, NDUFAB1, NDUFAF7, NDUFB3, NDUFS2, NDUFS3, NDUFS6, NDUFV1, OXCT1, PDSS1, Pka, PLIN5, PTGR1, SOD1, STXBP2, SUMO, TYRP1*	48	33
2.Molecular Transport, Endocrine System	*ACSF2, Alpha 1 antitrypsin, BOLA3, chymotrypsin, CLGN, CLIC6, ECH1, ESR1, FPGS, GLRX5, GMPPB, HDGFL3, HSF2BP, ISOC2, MPC2, MTERF2, MTMR12, NDUFAF5, NIT1, OGFOD3, PLXDC1, RAD51B, SAMD11, SCUBE2, SHROOM1, SLC25A20, SLC4A10, SLC4A11, SLC4A4/5/10/11, SLC4A5, SNN, SORD, THEM6, TTC9, XK*	45	32
3.Cell Cycle, Cell Morphology, Developmental Disorder	*ABHD12, ACSBG1, AES, CCDC8, CCDC92, CIRBP, Ck2, CRB1, DCLRE1B, EHBP1, FANCD2, GJC2, Gpm6b, HNRNPA1, INSR, Interferon alpha, ITM2C, KPNA2, MROH6, NAP1L5, NEK4, NMDA Receptor, PATJ, PIGS, PRDX1, Proinsulin, RIMKLB, RTL1, Shc, SLC22A23, Slc25a1, SLC5A6, Tdh, UBC, XPO1*	40	30
4.Nucleic Acid Metabolism, Small Molecule Biochemistry, Cellular Movement	*AK2, AK5, APCDD1, C2, CENPX, ETS, FAAP100, FAP, FH, GAMT, GREB1, IDH3B, IMPA2, IRX3, ITGBL1, KCTD2, LMO1, LXN, MASP1, MYC, NDPK, NMNAT2, NRIP3, PRSS23, SCPEP1, SCUBE3, Serine Protease, Smad2/3, Smad2/3-Smad4, SRD5A1, Steroid 5 alpha-Reductase, TAF1D, TKT, TSPAN7, ZNF217*	38	29
5.Amino Acid Metabolism, Small Molecule Biochemistry, Cancer	*AKAP4, ANP32A, ARHGAP22, BPHL, C1q, CALR3, DDAH2, DENND2D, DNA-methyltransferase, DOCK8, EGLN, FAT1, GPI, HDC, HIST2H4A, HISTONE, HOMER1, IFIT3, LZTFL1, MERTK, MSRA, NAB2, PARP, PARP3, PLA1A, RNASEH2A, RNF125, SAMSN1, SSR3, TCF23, TMEM107, TRIP13, TTC19, Vegf, YBX2*	38	29
6.Cellular Compromise, Inflammatory Response, Neurological Disease	*ABCB6, ACADS, BNIP3, CHAC2, CHI3L1, CORO2A, CRAT, DDHD2, DKK3, ENPEP, FCN1, Ferritin, GSDMB, HBD, HDL, hemoglobin, Iti, ITIH3, ITIH4, LCAT, LRRC8E, MAP1LC3, MLF1, NFKBIA, NT5DC1, ORM1, PIR, PROZ, PTX3, RGS3, SERPINB1, SPINT2, TF, TNFAIP6, VLDL-cholesterol*	38	29
7.Cellular Assembly and Organization, , Organ Development	*14-3-3, alcohol group acceptor phosphotransferase, Alpha Actinin, ATL2, ATP2B2, BCAP31, CACNA1G, Calmodulin, CAMK1G, CCND2, CD81, CDK18, DMPK, DMTN, F Actin, HIST1H2BO, Hsp90, KLF5, MAGED2, MAP1LC3A, MAP2K6, MDM1, SERINC2, SORCS2, SYNPO2, TMBIM6, TMEM160, TP53INP2, TSPAN4, TUBA1B, TUBB, TUBB4B, TUBB6, tubulin, WFS1*	36	28
8.Developmental Disorder, Hereditary Disorder, Metabolic Disease	*ABCC8, BDH1, cAMP-dependent protein kinase, CISD1, CMC2, COA3, COQ9, COX4I2, COX7B, cytochrome C, DHCR24, ETFDH, FUNDC2, HINT3, HPD, Mpt pore, MT-CO2, MT-CO3, NADH dehydrogenase, NDUFA13, NDUFA4, P2RX4, P2RX6, P38 MAPK, PFKFB2, POLDIP2, RIDA, Ryr, RYR1, SERCA, SLC25A4, TIMM23, TNFRSF6B, Tomm5, tubulin (family)*	34	27
9.Carbohydrate Metabolism, Small Molecule Biochemistry, Cell Morphology	*ACAT2, AKT1, ALDOA, ALDOC, ASB2, ATP5MF, ATP6V1E1, CGNL1, CRYZ, ENaC, GATM, Glycogen synthase, GYG2, GYS2, HADH, KCNMB1, KCNMB4, LSS, MAGIX, MRAS, MTA3, NCAPH, NCK, NCK1, Nuclear factor 1, Pak, PDGF-AA, PGD, Rab5, RASGRP2, Rho gdi, SH2B2, Troponin t, VAV3, WNK4*	32	26

10.Post-Translational Modification, Cell Morphology, Cellular Movement	*AKAP12, BAIAP2L1, Cdc2, CG, COA7, DUSP10, DUSP22, DUSP26, DUSP4, EPB41L4B, EPM2A, FSH, ion channel, IRS2, Lh, MAMLD1, Mapk, MEGF10, MOSPD1, PARD6B, Pde, PDE5A, PDF, Pka catalytic subunit, PTPase, PTPRD, PTPRS, ROBO1, ROBO2, SGTB, SIRPA, SMTNL2, TDRD5, TLN2, tyrosine kinase*	30	25
11.Cancer, Organismal Injury and Abnormalities, Carbohydrate Metabolism	*Actin, ADAM11, ADAM19, ADAMTS16, ADAMTS17, Alpha catenin, ANKRD35, BUD23, COX7A1, CSDC2, CXCL2, ERBB2, estrogen receptor, FBXO40, GSN, Integrin, IVNS1ABP, KIAA0408, LEP, LSR, Metalloprotease, Mmp, OGA, OGT, p85 (pik3r), PKNOX1, Rap1, RNA polymerase II, Rnr, SH3GL2, THRB, TNFRSF12A, VIM, ZNF106, ZNF280C*	30	25
12.Small Molecular Biochemistry, Metabolic Processes	*ABAT, ASGR1, COX20, Cytochrome bc1, cytochrome-c oxidase, ERK, FOXRED1, GK, Hif1, LDH (family), LDHB, LDHC, LDHD, MAP7, Mitochondrial complex 1, MT-ATP6, MT-CYB, MT-ND2, MT-ND3, MT-ND4L, Oas, Oxphos, PRDX3, RASD1, S100, SDHC, SIRT3, SMAD1/5, SULF1, Thioredoxin reductase, TXNRD2, UQCR10, UQCR11, UQCRC1, UQCRFS1*	28	24
13.Cell-To-Cell Signaling and Interaction, Protein Synthesis	*aldo, ASB16, ASB9, calpain, CAST, Cathepsin, CKB, creatine kinase, CTSD, CTSO, DPYSL3, ENO4, Enolase, Gap, Has, HCN2, HIST1H2BN, ITGA7, KIZ, Lysosomal Protease, MREG, NCS1, PFKFB, PGAM1, PHKA2, phosphatidylinositol-4,5-bisphosphate 3-kinase, PRUNE2, RAB3A, RASAL1, RCN3, RETSAT, SGCA, SRC, SYN2, Villin*	28	24
14.Carbohydrate Metabolism, Nucleic Acid Metabolism, Small Molecule Biochemistry	*ABCA7, ABCD1, ACAC, ANP32B, Ap2, ARHGEF37, ASMTL, ASS1, ATPase, BIN3, C/EBP, CSPG4, cytokine, DLX5, DQX1, EFS, ENTPD2, G6PD, HIST1H4J, HOXC8, Integrin alpha 4 beta 1, INTERLEUKIN, KCNK2, LDL-cholesterol, LIPA, MOG, nucleoside-triphosphatase, RNF128, SAA, SERPINF1, SMARCA1, SORT1, Sphk, TALDO1, VCAM1*	28	24
15.Hereditary Disorder, Organismal Injury and Abnormalities, Skeletal and Muscular Disorders	*ADTRP, ALDH, ALDH18A1, ALDH2, ALDH4A1, Angiotensin II receptor type 1, ANGPT2, BETA TUBULIN, Calcineurin protein(s), CaMKII, CDC34, CLMN, CRYAB, DNAJ, DNAJB5, FHL1, GUCY, GUCY2D, KALRN, KCNJ11, KIF1C, MYO1A, NADK, NFAT (complex), Nfat (family), NPR1, PITPNM1, Pp2b, PSPH, PVALB, Rac, S100B, SPTLC3, THBS2, TTN*	28	24
16.Lipid Metabolism, Small Molecule Biochemistry, Nucleic Acid Metabolism	*20s proteasome, ACLY, ACSS2, AMIGO3, AURK, Calcineurin A, CDC25A, Cdk, Cyclin B, EEF2K, ELOVL1, EPHX2, Gα12/13, HACD2, HSD17B, HSD17B11, HSD17B12, HSD17B14, IGF1, KLHL25, MSI2, Pdgf (complex), PDXP, PHLDB3, phosphatase, Pp2c, PPIP5K1, PPP5C, pyruvate kinase, RNF157, SESN1, SNCG, Srebp, TECR, TIGAR*	26	23
17.Cellular Assembly and Organization, Cell Cycle, Developmental Disorder	*Akt, Caveolin, CNTN1, Cox6c, Dynamin, EIF2B2, FKHR, GOT2, GSS, HEY1, IRS, LETM1, LRAT, MARCH2, MT-CO1, MYADM, N-type Calcium Channel, NMB, NOSTRIN, Notch, Nradd, PARM1, Pde4, PDGF-DD, PGF, PLP2, Presenilin, Secretase gamma, SLC16A1, STX12, STX17, Syntaxin, SYT17, VAMP8, Vegf Receptor*	25	22
18.Drug Metabolism, Protein Synthesis, Glutathione Depletion In Liver	*Adaptor protein 1, AIFM3, Aldose Reductase, CLU, CYP4V2, DBI, glutathione peroxidase, glutathione transferase, Glutathione-S-transferase, GST, Gst alpha, GSTA1, GSTA4, GSTM4, Gstt1, Gstt3, Gulo, HDL-cholesterol, HSPB6, Jnk, KLF11, Ldh (complex), MGST1, MGST2, OSBP2, PRNP, RASSF7, RBM47, Sod, SOD2, succinate dehydrogenase, TARSL2, TENM3, VLDL, VLDLR*	25	22
19.Connective Tissue Disorders, Organismal Injury and	*ADAMTS2, CHPT1, COL11A2, COL5A3, Collagen type I, Collagen type II, Collagen type III, Collagen type IV, CX3CL1, Eotaxin, F13A1, Fibrin, GPC1, GPIIB-IIIA, HABP4, Integrin alpha V beta 3, KCNC3,*	25	22

Abnormalities, Amino Acid Metabolism	*KCNC4, LAMB1, LAMC2, Laminin (complex), Laminin (family), Pkc(s), PLCD1, PLOD2, PMEPA1, Rab11, RGN, SLC1A1, SLC38A3, SLC6A9, Smad, SOX17, SSC5D, THY1*		
20.Connective Tissue Disorders, Organismal Injury and Abnormalities, Developmental Disorder	*ASGR2, CA10, CA11, CA4, Carbonic anhydrase, COL1A1, COL1A2, COL20A1, COL22A1, collagen, Collagen Alpha1, Collagen(s), ECHS1, Ecm, farnesyl transferase, FKRP, FNTB, JINK1/2, Kallikrein, LARP6, MMP15, MMP25, Mt-mmp, MTHFD1, Pdgf Ab, RAS, SLC4A4, SPARC, SRCIN1, Tgf beta, TGM3, TGM5, TIMP2, transglutaminase, UBXN11*	25	22
21.Lipid Metabolism, Small Molecule Biochemistry, Neurological Disease	*C2CD2, CAMK2N1, CCDC58, CX3CR1, ELAVL1, ELOVL1, FBXO8, FN3K, GLP1R, GLTP, GPR180, GSS, HACD2, ISOC1, KAZN, KDSR, LEPROT, LPAR3, MOB3B, MPV17L, MTFMT, PLEKHB1, PRPS2, PTEN, SETD9, SLC39A13, SLC46A3, SLC50A1, SPTSSA, SRD5A1, TCF7L2, TENT4A, TLNRD1, TMEM163, ZCCHC7*	25	22
22.Lipid Metabolism, Molecular Transport, Small Molecule Biochemistry	*1-acylglycerol-3-phosphate O-acyltransferase, Aconitase, AGPAT2, C8, CCDC3, CD3 group, CPT1, CPT2, CSRNP1, CYB5A, CYP, CYP19, DGAT2, DUB, elastase, GM2A, GOT, IL1B, Inflammasome (Nalp3, Asc, Casp1), ITM2B, LPCAT2, LPCAT3, MAC, MCUR1, METRNL, MFAP2, MGP, MIR124, PCYT2, RARRES2, RNASE1, SCD, SINHCAF, SLC25A25, Thrombospondin*	23	21
23.Gene Expression, Cell Morphology, Hematopoiesis	*Cbp/p300, CCDC85C, CDC42EP4, CEP89, DNA-directed RNA polymerase, DYNLRB2, ELL3, Gsk3, GTF2H5, Hat, HMGB2, Holo RNA polymerase II, IFN alpha/beta, IFN Beta, IFN type 1, Ifnar, Ikk (family), IRAK, IRF, IRF8, KIF7, MAT2A, POLR2E, POLR3G, POLR3H, POLR3K, PPP1R2, SEPT6, SH3BGRL2, SNAPC1, SNAPC5, TH1 Cytokine, Tlr, TLR8, TRAF3IP2*	23	21
24.Lipid Metabolism, Nucleic Acid Metabolism, Small Molecule Biochemistry	*26s Proteasome, ADRB, AKR7A2, AMPK, Ap1, ARRDC2, DDIT4, EIF4G1, FASN, HIF1A, HIST1H2AC, Hsp27, IL-1R, LAMTOR1, MTORC1, NQO1, ORC1, p70 S6k, PDGF BB, PDHA1, PDZRN3, PEBP1, PI3K (family), PLA2, Pld, PPT1, Ras homolog, SDHA, SLC1A5, TBKBP1, TXN2, TXNIP, Ubiquitin, UCHL1, UCHL3*	23	21
25.Cell Signaling, Nucleic Acid Metabolism, Cell-To-Cell Signaling and Interaction	*ACKR3, ADCY, Beta Arrestin, CDS1, CERS6, Clathrin, CX3CR1, Endothelin, FFAR4, G protein, G protein beta gamma, G-protein beta, Gpcr, GPER1, GPR156, GPR21, GPR37, GPR37L1, GPRC5A, Gs-coupled receptor, HCAR3, HCRTR1, HMOX2, MCHR1, OPRL1, P110, P2RY2, protein phosphatase, Relaxin, Rgs, RGS1, S1PR3, Sfk, VIPR2, voltage-gated calcium channel*	21	20

Supplementary Table 4: Genes identified as up-regulated in visceral adipose of heifer calves offered a High plane of nutrition compared to a Moderate plane of nutrition for the first 21 weeks of life and mapped in Ingenuity Pathway Analysis (IPA).

**Pathway name**	**adj. p-value**	**Genes**
Oxidative Phosphorylation	3.98E-29	*ATP5MF, COX4I2 , Cox6c, COX7A1, COX7B, CYB5A, MT-ATP6, MT-CO1, MT-CO2, MT-CO3, MT-CYB, MT-ND1, MT-ND2, MT-ND3, MT-ND4, MT-ND4L, MT-ND5, NDUFA1, NDUFA10, NDUFA13, NDUFA2, NDUFA4, NDUFAB1, NDUFB3, NDUFS2, NDUFS3, NDUFS6, NDUFV1, SDHA, SDHC, UQCR10, UQCR11, UQCRC1, UQCRFS1*
Mitochondrial Dysfunction	1.58E-27	*ATP5MF, COX4I2, Cox6c, COX7A1, COX7B, CYB5A, MT-ATP6, MT-CO1, MT-CO2, MT-CO3, MT-CYB, MT-ND1, MT-ND2, MT-ND3, MT-ND4, MT-ND4L, MT-ND5, NDUFA1, NDUFA10, NDUFA13, NDUFA2, NDUFA4, NDUFAB1, NDUFB3, NDUFS2, NDUFS3, NDUFS6, NDUFV1, PDHA1, PRDX3, SDHA, SDHC, SOD2, TXN2, TXNRD2, UQCR10, UQCR11, UQCRC1, UQCRFS1*
Sirtuin Signaling Pathway	1.58E-20	*ACLY, ACSS2, ARNTL, G6PD, GOT2, LDHB, LDHC, LDHD, MAP1LC3A, MT-ATP6, MT-CYB, MT-ND1, MT-ND2, MT-ND3, MT-ND4, MT-ND4L, MT-ND5, NDUFA1, NDUFA10, NDUFA13, NDUFA2, NDUFA4, NDUFAB1, NDUFB3, NDUFS2, NDUFS3, NDUFS6, NDUFV1, NQO1, PCK1, PDHA1, PGAM1, SDHA, SDHC, SIRT3, SLC25A4, SOD1, SOD2, TIMM23, Tomm5, UQCRFS1*
Superoxide Radicals Degradation	1.86E-05	*NQO1, SOD1, SOD2, TYRP1*
Pentose Phosphate Pathway	5.37E-05	*G6PD, PGD, TALDO1, TKT*
Valine Degradation I	5.89E-05	*ABAT, ACADSB, ECHS1, HIBADH, SDS*
Glutathione-mediated Detoxification	8.13E-05	*GSTA1, GSTA4, GSTM4, Gstt1, Gstt3, MGST1*
Xenobiotic Metabolism AHR Signaling Pathway	0.000155	*ALDH18A1, ALDH2, ALDH4A1, GSTA1, GSTA4, GSTM4, IL1B, MGST1, NQO1*
Gluconeogenesis I	0.000288	*ALDOA, ALDOC, GPI, ME1, PGAM1*
Isoleucine Degradation I	0.000417	*ACADSB, ACAT2, ECHS1, SDS*
NRF2-mediated Oxidative Stress Response	0.00049	*AKR7A2, CDC34, DNAJB5, GSTA1, GSTA4, GSTM4, MAP2K6, MGST1, MRAS, NQO1, PRDX1, SOD1, SOD2*
Superpathway of Cholesterol Biosynthesis	0.00049	*ACAT2, DHCR24, IDI1, LSS, NSDHL*
Aryl Hydrocarbon Receptor Signaling	0.000513	*ALDH18A1, ALDH2, ALDH4A1, CTSD, ESR1, GSTA1, GSTA4, GSTM4, IL1B, MGST1, NQO1*
Ethanol Degradation II	0.000794	*ACSS2, ADH6, ADHFE1, ALDH2, ALDH4A1*
Fatty Acid β-oxidation I	0.000794	*ACSBG1, ECHS1, HADH, IVD, SDS*
Sucrose Degradation V (Mammalian)	0.000933	*ALDOA, ALDOC, KHK*
Ketolysis	0.001318	*ACAT2, BDH1, OXCT1*
CDP-diacylglycerol Biosynthesis I	0.001514	*AGPAT2, CDS1, GPAM, LPCAT2*
Ethanol Degradation IV	0.001778	*ACSS2, ALDH2, ALDH4A1, TYRP1*

LXR/RXR Activation	0.002042	*CLU, ECHS1, FASN, HADH, IL1B, ORM1, SCD, SERPINF1, TF*
Phosphatidylglycerol Biosynthesis II (Non-plastidic)	0.002089	*AGPAT2, CDS1, GPAM, LPCAT2*
TCA Cycle II (Eukaryotic)	0.002089	*FH, IDH3B, SDHA, SDHC*
LPS/IL-1 Mediated Inhibition of RXR Function	0.002399	*ACOX1, ACOX2, ACSBG1, ALDH18A1, ALDH2, ALDH4A1, CPT2, CRAT, GSTA1, GSTA4, GSTM4, IL1B, MGST1*
Intrinsic Prothrombin Activation Pathway	0.002754	*COL1A1, COL1A2, COL5A3, F13A1, KLK7*
Glycolysis I	0.002818	*ALDOA, ALDOC, GPI, PGAM1*
Cholesterol Biosynthesis I	0.002951	*DHCR24, LSS, NSDHL*
Cholesterol Biosynthesis II (via 24, 25-dihydrolanosterol)	0.002951	*DHCR24, LSS, NSDHL*
Cholesterol Biosynthesis III (via Desmosterol)	0.002951	*DHCR24, LSS, NSDHL*
Pentose Phosphate Pathway (Oxidative Branch)	0.00309	*G6PD, PGD*
Phenylethylamine Degradation I	0.00309	*ALDH2, AOC2*
Phenylalanine Degradation IV (Mammalian, via Side Chain)	0.003715	*ALDH2, GOT2, HPD*
Stearate Biosynthesis I (Animals)	0.00537	*ACSBG1, DHCR24, FASN, PPT1, TECR*
Androgen Biosynthesis	0.005495	*GSTA1, HSD17B14, SRD5A1*
Pyruvate Fermentation to Lactate	0.007586	*LDHB, LDHC*
Ceramide Biosynthesis	0.007586	*KDSR, SPTLC3*
Pentose Phosphate Pathway (Non-oxidative Branch)	0.007586	*TALDO1, TKT*
Rapoport-Luebering Glycolytic Shunt	0.007586	*PGAM1, TIGAR*
Superpathway of Geranylgeranyldiphosphate Biosynthesis I (via Mevalonate)	0.007762	*ACAT2, FNTB, IDI1*
Noradrenaline and Adrenaline Degradation	0.008511	*ADH6, ADHFE1, ALDH2, ALDH4A1*
HIF1α Signaling	0.008511	*CCNG2, FOXP3, GPI, LDHB, LDHC, MAP2K6, MMP15, MMP25, MRAS, PGF, TF*
Oxidative Ethanol Degradation III	0.00912	*ACSS2, ALDH2, ALDH4A1*
Atherosclerosis Signaling	0.00955	*CLU, COL1A1, COL1A2, COL5A3, IL1B, ORM1, PLA2G5, VCAM1*
Glycogen Biosynthesis II (from UDP-D-Glucose)	0.010471	*EPM2A, GYS2*
Glutathione Redox Reactions I	0.017378	*GSTA1, Gstt1, MGST1*
Triacylglycerol Biosynthesis	0.018621	*AGPAT2, DGAT2, GPAM, LPCAT2*
Serotonin Degradation	0.019498	*ADH6, ADHFE1, ALDH2, ALDH4A1, Sult1a1*
Acute Phase Response Signaling	0.025119	*CRABP2, HMOX2, IL1B, MAP2K6, MRAS, ORM1, SERPINF1, SOD2, TF*
Ketogenesis	0.025704	*ACAT2, BDH1*

FXR/RXR Activation	0.027542	*CLU, FASN, IL1B, ORM1, SERPINF1, TF, VLDLR*
Dendritic Cell Maturation	0.028184	*CD1A, COL1A1, COL1A2, COL5A3, HLA-DMB, IL1B, IL23A, IRF8, LEP*
3-phosphoinositide Degradation	0.029512	*CDC25A, DUSP10, EPHX2, HACD2, INPP5K, PDXP, PPIP5K1, SIRPA*
Dopamine Degradation	0.031623	*ALDH2, ALDH4A1, Sult1a1*
Toll-like Receptor Signaling	0.031623	*IL1B, MAP2K6, TLR8, TRAF4, UBC*
Oleate Biosynthesis II (Animals)	0.035481	*CYB5A, SCD*
Mevalonate Pathway I	0.040738	*ACAT2, IDI1*
Estrogen Receptor Signaling	0.043652	*EIF2B2, ESR1, GPER1, LEP, MMP15, MMP25, MRAS, MT-ATP6, MT-CYB, PGF, SDHC, SOD2, UQCRFS1*
TR/RXR Activation	0.045709	*FASN, ME1, PCK1, THRB, THRSP*
D-myo-inositol (1, 4, 5, 6)-Tetrakisphosphate Biosynthesis	0.047863	*CDC25A, DUSP10, EPHX2, HACD2, PDXP, PPIP5K1, SIRPA*
D-myo-inositol (3, 4, 5, 6)-tetrakisphosphate Biosynthesis	0.047863	*CDC25A, DUSP10, EPHX2, HACD2, PDXP, PPIP5K1, SIRPA*
Xenobiotic Metabolism General Signaling Pathway	0.048978	*GSTA1, GSTA4, GSTM4, MAP2K6, MGST1, MRAS, NQO1*

Supplementary Table 5: Genes identified as down-regulated in visceral adipose of heifer calves offered a High plane of nutrition compared to a Moderate plane of nutrition for the first 21 weeks of life and mapped in Ingenuity Pathway Analysis (IPA).

**Pathway name**	**adj. p-value**	**Genes**
STAT3 Pathway	3.63E-06	*CISH, FGFR3, IGF1, IL20RA, IL6R, INSR, MYC, RASD1, SOCS1, SOCS2, SRC, VEGFA*
14-3-3-mediated Signaling	1.17E-05	*AKT1, MAPT, PLCD1, PLCH2, RASD1, SRC, TUBA1B, TUBB, TUBB4B, TUBB6, VIM*
Gap Junction Signaling	0.000631	*AKT1, GRIK2, NPR1, PLCD1, PLCH2, RASD1, SRC, TUBA1B, TUBB, TUBB4B, TUBB6*
Angiopoietin Signaling	0.001778	*AKT1, ANGPT2, GRB7, NCK1, NFKBIA, RASD1*
Axonal Guidance Signaling	0.002239	*ADAM11, AKT1, ERBB2, IGF1, KALRN, KIF7, NCK1, NTN5, PLCD1, PLCH2, RASD1, RGS3, ROBO2, TUBA1B, TUBB, TUBB4B, TUBB6, VEGFA*
JAK/Stat Signaling	0.002455	*AKT1, CISH, RASD1, SOCS1, SOCS2, STAT6*
Role of JAK2 in Hormone-like Cytokine Signaling	0.00257	*IRS2, SH2B2, SOCS1, SOCS2*
Serine Biosynthesis	0.002818	*DUSP26, PSPH*
Citrulline-Nitric Oxide Cycle	0.002818	*ASL, ASS1*
Protein Kinase A Signaling	0.003802	*AKAP4, DUSP22, DUSP26, DUSP4, NFKBIA, PHKA2, PLCD1, PLCH2, PTGS2, PTPRD, PTPRS, RYR1, TNNI2, TNS2, TTN*
Acute Phase Response Signaling	0.00389	*AKT1, C2, IL6R, ITIH3, ITIH4, NFKBIA, RASD1, SOCS1, SOCS2*
Arginine Biosynthesis IV	0.004169	*ASL, ASS1*
Urea Cycle	0.004169	*ASL, ASS1*
Phospholipases	0.005012	*LCAT, PLA1A, PLCD1, PLCH2, PLD5*
Superpathway of Serine and Glycine Biosynthesis I	0.005754	*DUSP26, PSPH*
IL-6 Signaling	0.006026	*AKT1, IL6R, NFKBIA, RASD1, SOCS1, TNFAIP6, VEGFA*
Remodeling of Epithelial Adherens Junctions	0.006166	*SRC, TUBA1B, TUBB, TUBB4B, TUBB6*
IL-23 Signaling Pathway	0.006607	*AKT1, HIF1A, NFKBIA, RORC*
p70S6K Signaling	0.006761	*AKT1, EEF2K, MAPT, PLCD1, PLCH2, RASD1, SRC*
IL-8 Signaling	0.007586	*AKT1, ANGPT2, CCND2, PLD5, PTGS2, RASD1, RHOB, SRC, VEGFA*
ERK5 Signaling	0.007762	*AKT1, MYC, RASD1, SGK1, SRC*
Role of Macrophages, Fibroblasts and Endothelial Cells in Rheumatoid Arthritis	0.007943	*AKT1, DKK3, IL6R, MYC, NFKBIA, PLCD1, PLCH2, RASD1, SOCS1, SRC, TRAF3IP2, VEGFA*
Estrogen-Dependent Breast Cancer Signaling	0.00871	*AKT1, HSD17B11, IGF1, RASD1, SRC*
HIF1α Signaling	0.008913	*AKT1, CAMK1G, HIF1A, IGF1, IL6R, RASD1, RORC, VEGFA, VIM*
IGF-1 Signaling	0.008913	*AKT1, IGF1, IRS2, RASD1, SOCS1, SOCS2*
Neuregulin Signaling	0.009333	*AKT1, ERBB2, GRB7, MYC, RASD1, SRC*

PI3K Signaling in B Lymphocytes	0.00955	*AKT1, IRS2, MALT1, NFKBIA, PLCD1, PLCH2, RASD1*
Erythropoietin Signaling	0.01	*AKT1, CCND2, CLCF1, HIF1A, IRS2, NFKBIA, RASD1, SRC*
mTOR Signaling	0.010233	*AKT1, DDIT4, HIF1A, INSR, PLD5, PRR5L, RASD1, RHOB, VEGFA*
Opioid Signaling Pathway	0.010233	*AKT1, CAMK1G, MYC, NFKBIA, OPRL1, RASD1, RGS1, RGS3, RYR1, SRC*
Integrin Signaling	0.01122	*AKT1, GRB7, GSN, NCK1, RASD1, RHOB, SRC, TSPAN4, TTN*
BEX2 Signaling Pathway	0.011482	*AKT1, ERBB2, HIF1A, NFKBIA, VEGFA*
Epithelial Adherens Junction Signaling	0.015849	*AKT1, RASD1, SRC, TUBA1B, TUBB, TUBB4B, TUBB6*
Endothelin-1 Signaling	0.015849	*LCAT, MYC, PLCD1, PLCH2, PLD5, PTGS2, RASD1, SRC*
GP6 Signaling Pathway	0.016596	*AKT1, COL11A2, COL20A1, COL22A1, LAMB1, RASGRP2*
ILK Signaling	0.016596	*AKT1, HIF1A, IRS2, MYC, PTGS2, RHOB, VEGFA, VIM*
IL-9 Signaling	0.018197	*CISH, IRS2, SOCS2*
ErbB2-ErbB3 Signaling	0.025119	*AKT1, ERBB2, MYC, RASD1*
Superpathway of Citrulline Metabolism	0.026303	*ASL, ASS1*
VEGF Signaling	0.027542	*AKT1, HIF1A, RASD1, SRC, VEGFA*
Adipogenesis pathway	0.027542	*AKT1, FGFR3, HIF1A, KLF5, PER2, TXNIP*
Role of JAK1 and JAK3 in γc Cytokine Signaling	0.0302	*IRS2, RASD1, SOCS1, STAT6*
Agrin Interactions at Neuromuscular Junction	0.031623	*ERBB2, LAMB1, RASD1, SRC*
Growth Hormone Signaling	0.033113	*IGF1, IGFALS, SOCS1, SOCS2*
Insulin Receptor Signaling	0.033113	*AKT1, INSR, IRS2, NCK1, RASD1, SGK1*
Pyrimidine Ribonucleotides Interconversion	0.034674	*AK5, HNRNPA1, SMARCA1*
Hypoxia Signaling in the Cardiovascular System	0.038019	*AKT1, HIF1A, NFKBIA, VEGFA*
EIF2 Signaling	0.038905	*AKT1, HNRNPA1, INSR, MYC, RASD1, RPL3, RPLP0, VEGFA*
Pyrimidine Ribonucleotides De Novo Biosynthesis	0.038905	*AK5, HNRNPA1, SMARCA1*
IL-15 Signaling	0.039811	*AKT1, MYC, RASD1, STAT6*
Regulation Of The Epithelial Mesenchymal Transition By Growth Factors Pathway	0.042658	*AKT1, ERBB2, FGFR3, IL6R, PARD6B, RASD1, VIM*
Phagosome Maturation	0.045709	*CTSO, DYNLRB2, TUBA1B, TUBB, TUBB4B, TUBB6*
Systemic Lupus Erythematosus In B Cell Signaling Pathway	0.047863	*AKT1, CCND2, CLCF1, IL6R, MALT1, MYC, RASD1, RASGRP2, SRC*
IL-17 Signaling	0.047863	*AKT1, PTGS2, RASD1, TRAF3IP2*
